# Durable Lithium Metal Anodes Enabled by {110}‐Textured Epitaxy on a LiF@Ag Commensurate Heterostructure

**DOI:** 10.1002/advs.202510886

**Published:** 2025-08-07

**Authors:** Liming Zhang, Xuemin Wang, Xiaotong Sun, Haobo Yang, Xinyi Miao, Zifeng Gu, Chuanzhong Chen, Cheng Hu

**Affiliations:** ^1^ Key Laboratory for Liquid‐Solid Structural Evolution and Processing of Materials (Ministry of Education) School of Materials Science and Engineering Shandong University Jinan Shandong 250061 China; ^2^ Shandong Engineering & Technology Research Center for Superhard Materials Shandong University Jinan Shandong 250061 China; ^3^ Shenzhen Research Institute of Shandong University Shenzhen Guangdong 518057 China

**Keywords:** commensurate heterostructures, crystallographic textures, lithium fluoride, lithium metal anodes, silver

## Abstract

Lithium metal anodes (LMAs) hold great promise for high‐energy storage, but their practical application is hindered by challenges such as Li dendrite growth and unstable solid electrolyte interphase. Designing heteroepitaxial substrates to guide {110}‐textured Li growth is a promising strategy to suppress dendrite formation and parasitic reactions. LiF and Ag are potential candidates owing to their low lattice mismatch with Li, whilst the former lacks sufficient lithiophilicity and the latter suffers from Li‐Ag alloying. A LiF@Ag commensurate heterostructure composed of Ag nanoparticles (NPs) uniformly grown on 2D LiF layers is hence proposed, where the composition pinning effect prevents Li‐Ag alloying and stabilizes the lithiophilic Ag NPs upon Li plating. Meanwhile, Ag contributes to a significantly reduced Li adatom diffusion barrier compared with LiF, providing a synergistic effect of low lattice mismatch and improved interfacial dynamics to promote Li heteroepitaxy. Ultra‐dense and dendrite‐free Li heteroepitaxial deposition with prominent {110} texture is achieved, which enables improved coulombic efficiency, enhanced high‐rate capability, and prolonged cycle life in Li metal full cells with a low negative‐to‐positive ratio. The results demonstrate that constructing commensurate heterostructures offers a new synergistic strategy to regulate both deposition thermodynamics and kinetics toward high‐performance {110}‐textured LMAs.

## Graphical abstract

Utilizing their commensurate lattices, a LiF@Ag heterostructure is fabricated for Li epitaxy, featuring Ag nanoparticles grown on 2D LiF layers. The composition pinning effect inhibits Li‐Ag alloying and maintains the high lithiophilicity and fast adatom migration associated with Ag. Enhanced {110}‐textured Li deposition with extended cycle life is achieved through the synergistic control of deposition thermodynamics and kinetics.

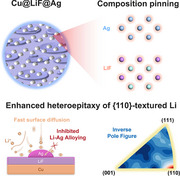

## 1 Introduction

Lithium (Li) metal anodes (LMAs) offer significant advantages for high‐energy battery systems, including a high theoretical specific capacity of 3860 mAh g^−1^ and the most negative standard electrode potential of −3.04 V *vs*. the standard hydrogen electrode.^[^ ^1^ ^]^ However, their practical deployment is fundamentally challenged by persistent interfacial issues, including the uncontrolled growth of Li dendrites and parasitic reactions with the electrolyte.^[^ ^1g,k^ ^]^ The dendritic deposition of Li on conventional copper (Cu) current collectors in carbonate or ether‐based electrolytes imposes safety hazards of internal short circuits and thermal runaway.^[^ ^2^ ^]^ The high chemical reactivity of metallic Li triggers continuous electrolyte decomposition, resulting in an unstable solid electrolyte interphase (SEI).^[^ ^3^ ^]^ This dynamic interfacial evolution leads to low Coulombic efficiency (CE)^[^ ^4^ ^]^ during extended cycling and the continuous depletion of Li and electrolyte inventory, which is particularly detrimental in high‐energy‐density configurations with low negative‐to‐positive (N/P) ratios.^[^ ^5^ ^]^ Therefore, constructing stable and efficient Li metal anodes remains a critical bottleneck for advancing the LMA technology.

Recent research has demonstrated that controlling the crystallographic texture of Li deposition offers a promising pathway to mitigate dendrite proliferation and parasitic reactions with the electrolyte.^[^ ^6^ ^]^ Due to the inherently low diffusion barrier and enhanced lateral diffusion kinetics, deposition on the Li(110) plane can facilitate 2D Li growth with a planar morphology, effectively suppressing undesirable dendritic Li growth.^[^ ^7^ ^]^ Meanwhile, the Li(110) plane, recognized as the most densely packed plane within the body‐centered cubic (BCC) lattice, exhibits reduced surface energy and a lower propensity for parasitic reactions.^[^ ^6d^ ^]^ Current approaches to {110}‐textured Li metal anodes, where the Li(110) plane is preferably aligned parallel to the electrode surface, primarily focus on thermodynamic driving forces, including the minimization of surface, interface, and strain energy. Zhao et al. reported that increasing the plating capacity to high values leads to a transition to {110}‐textured dense deposits due to surface energy minimization.^[^ ^6d^ ^]^ Li et al. employed self‐assembled reduced graphene oxide (rGO) on Cu to guide the planar growth of Li, where the low lattice mismatch of rGO with Li(110) minimizes the interfacial energy.^[^ ^6b^ ^]^ The textured anode maintained a high CE of ≈98% for 300 cycles at a current density of 2.0 mA cm^−2^. Tan et al. reported a strain‐energy‐driven method to control the {110} texture evolution by using accumulative roll bonding (ARB),^[^ ^6k^ ^]^ where lower overpotential, uniform Li deposition, and enhanced cycling stability were achieved simultaneously. Among these methods, designing a low‐lattice‐mismatch heteroepitaxial substrate to guide (110)‐oriented Li growth is of particular interest as it provides a straightforward method to {110}‐textured Li anodes through routine cell charging.^[^ ^6m^ ^]^

The widely used Cu current collector cannot serve as a Li heteroepitaxial substrate due to its large lattice mismatch.^[^ ^8^ ^]^ Modifying Cu with materials possessing a high supercell matching with Li can be an optimal strategy, as demonstrated by Li et al. using rGO.^[^ ^6b^ ^]^ However, the electrodeposition and crystal growth of Li on a foreign substrate involves other factors, including chemical compatibility^[^ ^9^ ^]^ and adatom adsorption and diffusion, beyond the simple geometric matching of their lattices.^[^ ^10^ ^]^ The impact of interfacial kinetic processes such as Li adatom diffusion and alloying reactions, which are particularly relevant to Li heteroepitaxy, remains unexplored despite being critical to the obtained Li morphology and the durability of the modified substrate along repeated charge and discharge.^[^ ^11^ ^]^

Recent advances in LMA interface modification offer a wide range of materials that can be chosen to regulate the complex interplay among lattice mismatch, surface diffusion, and structural stability.^[^ ^12^ ^]^ Lithium fluoride (LiF) and silver (Ag) are potential candidates to serve as a heteroepitaxial buffer layer owing to the proper lattice matching of their (110) planes with both Cu(110) and Li(110) (Table S1, Supporting Information).^[^ ^8^ ^]^ As a key component of the SEI, LiF possesses high mechanical strength and chemical stability that prevents undesirable reactions with Li and the electrolyte.^[^ ^13^ ^]^ Meanwhile, Ag exhibits a high lithiophilicity and a low surface diffusion barrier for Li adatoms.^[^ ^14^ ^]^ However, the lithiophilicity of LiF is less competitive than Ag, whilst the propensity of Ag to readily alloy with Li raises concerns about its durability. A closer look of LiF and Ag suggests that they satisfy a stricter commensurate relationship (lattice mismatch of only 0.5%), indicating that Ag grown on LiF will inherit a composition pinning effect from the latter,^[^ ^15^ ^]^ which will improve the interfacial stability of Ag with deposited Li while maintaining its merits of high lithiophilicity and facilitated surface diffusion. This is expected to act as a synergistic strategy for controlling the preferred orientation of Li deposition, wherein the construction of commensurate interfaces simultaneously regulates crystallographic constraints and deposition kinetics during textured heteroepitaxial growth.

The study reported here confirms the limitations of pure LiF or Ag as heteroepitaxial substrates for {110}‐textured Li deposition. The inadequate lithiophilicity and surface diffusion of LiF(110) induce uneven Li nucleation with a large overpotential, resulting in a less pronounced {110} texture despite presenting a low lattice mismatch with Li(110). Meanwhile, Ag exhibits no epitaxial ability of Li due to the Li‐Ag alloying process, which leads to structural pulverization and rapid deactivation of the lithiophilic sites.^[^ ^16^ ^]^ A LiF@Ag commensurate heterostructure composed of Ag nanoparticles (NPs) uniformly grown on 2D LiF layers is hence prepared using pulsed laser deposition (PLD). Li deposition and cyclic voltammetry measurements indicate that this commensurate heterostructure prevents Li‐Ag alloying, thereby ensuring the structural integrity of the Ag NPs during repeated charge and discharge. Furthermore, the low strain energy at the LiF and Ag interface maintains the lithiophilicity and low surface diffusion barrier, working together with the low lattice mismatch to strengthen the {110} texture while allowing an ultra‐dense, dendrite‐free morphology. The resulting {110}‐textured heteroepitaxial growth of Li delivers stable plating/stripping with an improved average coulombic efficiency (ACE) of 99.5%. LiFePO_4_ (LFP) full cells with a low N/P ratio of 0.49 exhibit excellent high‐rate capability up to 5 C and a high capacity retention of 80.5% over 180 charge and discharge cycles at 0.5 C. Preparing commensurate heterostructures as the Li heteroepitaxial substrate offers a new synergistic strategy that regulates deposition thermodynamics and kinetics simultaneously toward highly {110}‐textured Li anodes.

## 2 Results and Discussion

### 2.1 Modification of the Cu Substrate

**Figure 1:**
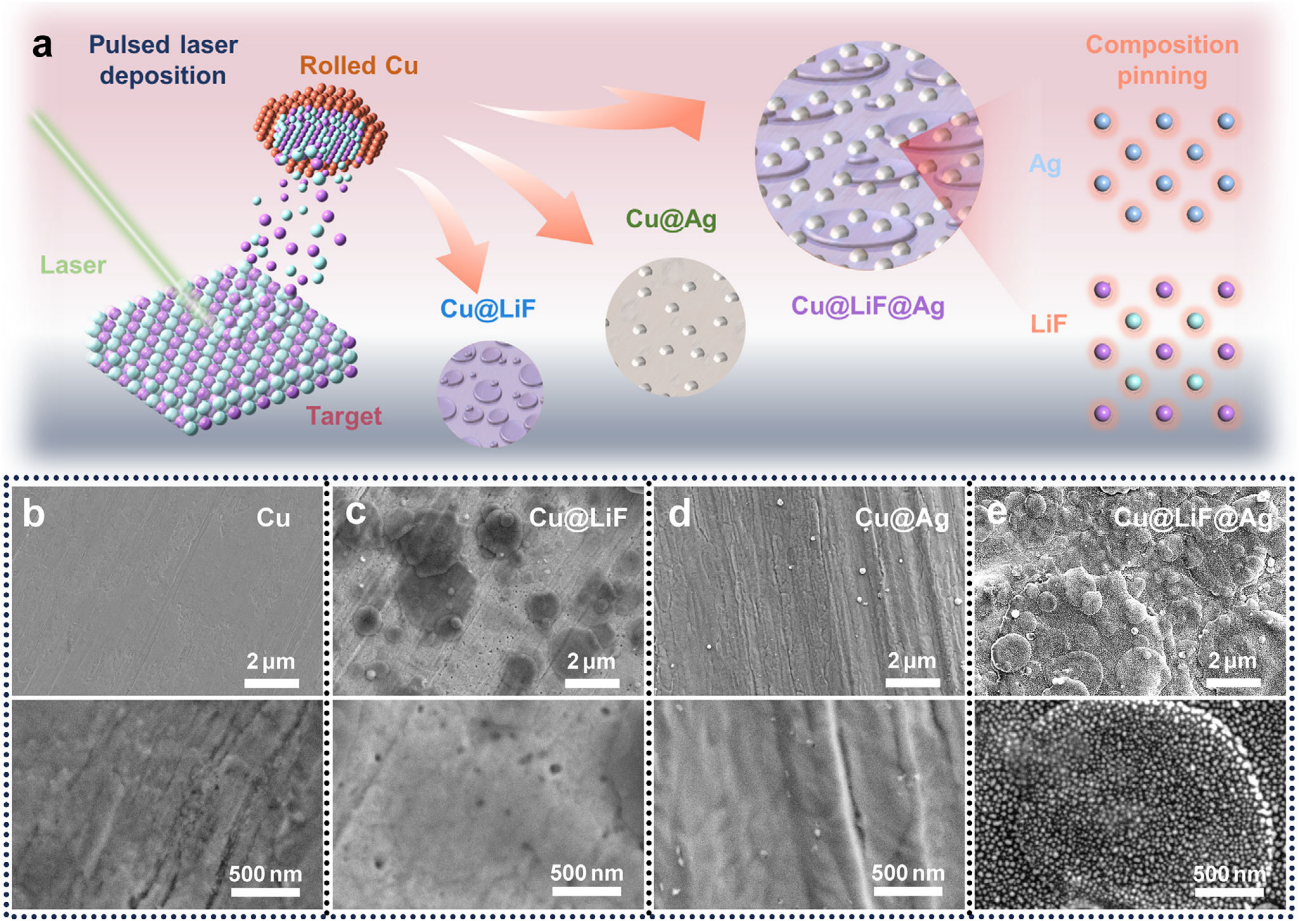
Modification of the Cu substrate with LiF, Ag, and the LiF@Ag commensurate heterostructure. a) Schematic illustration for the modification process using pulsed laser deposition. The LiF@Ag commensurate heterostructure was prepared by first depositing a LiF layer on the Cu substrate, followed by the growth of Ag NPs on LiF, where the composition pinning effect improves the interfacial stability of Ag with Li. SEM morphologies of b) the pristine Cu substrate and those modified by c) LiF only (denoted as Cu@LiF), d) Ag only (denoted as Cu@Ag), and e) the commensurate LiF@Ag heterostructure (denoted as Cu@LiF@Ag).

As schematically shown in **Figure** **1**a, the Cu substrate was modified with LiF, Ag, and the LiF@Ag commensurate heterostructure using PLD. This technique was chosen for its ability to achieve layer‐by‐layer control using multiple targets, with defined amounts of material transferred onto the substrate enabled by controlling the deposition time.^[^ ^17^ ^]^ The rolled Cu substrate, textured with the Cu(110) plane parallel to the surface (Figure S1, Supporting Information), acts as a template for the arriving LiF or Ag to coalesce and self‐assemble into a thin film with a preferred orientation inherited from the Cu substrate. The Cu substrate modified by the LiF@Ag commensurate heterostructure (denoted as Cu@LiF@Ag) was prepared by first depositing a LiF layer, followed by the growth of Ag NPs on LiF by controlling the deposition time. Pure LiF or Ag was also employed to modify the Cu substrate for comparison, referred to as Cu@LiF and Cu@Ag, respectively. The loading of LiF and Ag on the modified substrates is confirmed by grazing incidence X‐ray diffraction (GIXRD) and energy dispersive spectroscopy (EDS) associated with scanning electron microscopy (SEM) (Figures S2 and S3, Supporting Information).

The resulting morphologies after modification exhibit significant variations, indicative of the interplay between substrate texture, deposition parameters, and material compatibility. SEM images reveal that the pristine Cu substrate is characterized by rolling strips (Figure 1b), resulting from grain deformation and surface rumpling during the rolling process.^[^ ^18^ ^]^ The LiF layer grown on Cu (Figure 1c) consists of stacked and planar‐grown flakes, resulting from droplet splashing.^[^ ^19^ ^]^ The visible rolling strips in the high‐magnification image indicate a low thickness of the LiF layers, which ensures sufficient electron tunneling for the following electrodeposition of Li. The deposition of Ag on Cu presents a markedly different morphology. Leveraging the shared face‐centered cubic (FCC) structure and a modest lattice mismatch (MCIA = 73.4), a more uniform Ag growth was observed on Cu (Figure 1d). This epitaxial compatibility facilitates layer‐by‐layer growth, effectively filling the surface microcracks induced by the rolling process of Cu, thereby producing a smoother surface morphology.

In contrast, Cu@LiF@Ag exhibits a unique morphology, with Ag NPs uniformly distributed on the LiF layer (Figure 1e). The morphological evolution during metal film growth follows a progression from isolated isometric islands to elongated islands, multi‐connected non‐permeable islands, and finally to percolating metal films.^[^ ^20^ ^]^ By controlling the deposition time of Ag to 10 min, the growth of Ag was terminated at the stage of isolated isometric islands on LiF, resulting in ultra‐fine Ag NPs with sizes ranging from 20 to 30 nm. The transition to elongated islands with an extended deposition time of 15 min further supports this evolutionary process (Figure S4, Supporting Information).

### 2.2 Texture and Morphology of the Li Deposition Layer

The crystallography of deposited Li on the pristine Cu, Cu@LiF, Cu@Ag, and Cu@LiF@Ag substrates was first examined using X‐ray diffraction (XRD) with the Bragg‐Brentano optics. Li was deposited onto the substrates using a current density of 1 mA cm^−2^ to a capacity of 10 mAh cm^−2^. The XRD patterns are shown in Figure S5a (Supporting Information), and the relative texture coefficient (RTC) was calculated to act as a semi‐quantitative measure of crystallographic orientation. As shown in Figure S5b (Supporting Information), Cu@LiF@Ag presents the highest RTC for the (110) plane, indicating an enhanced {110} texture of the deposited Li. However, XRD measurements using the Bragg‐Brentano optics only capture crystal lattice planes oriented parallel to the sample surface. The complete crystallographic orientation of the deposited Li was hence examined by XRD pole figure analysis to further reveal the impact of interfacial dynamics in controlling (110)‐oriented heteroepitaxy. Pole figure analysis was performed by varying the azimuthal (*ϕ*) and tilting (*ψ*) angles to obtain a hemispherical intensity map of crystallographic plane orientations as a function of *ϕ* and *ψ*.^[^ ^6a^ ^]^ A stereographic projection then produces a pole figure plot, where the pole density intensity *I* (*ϕ*, *ψ*) represents the orientation of the measured crystallographic plane against the sample coordinate system (Figure S6, Supporting Information).^[^ ^21^ ^]^

**Figure 2:**
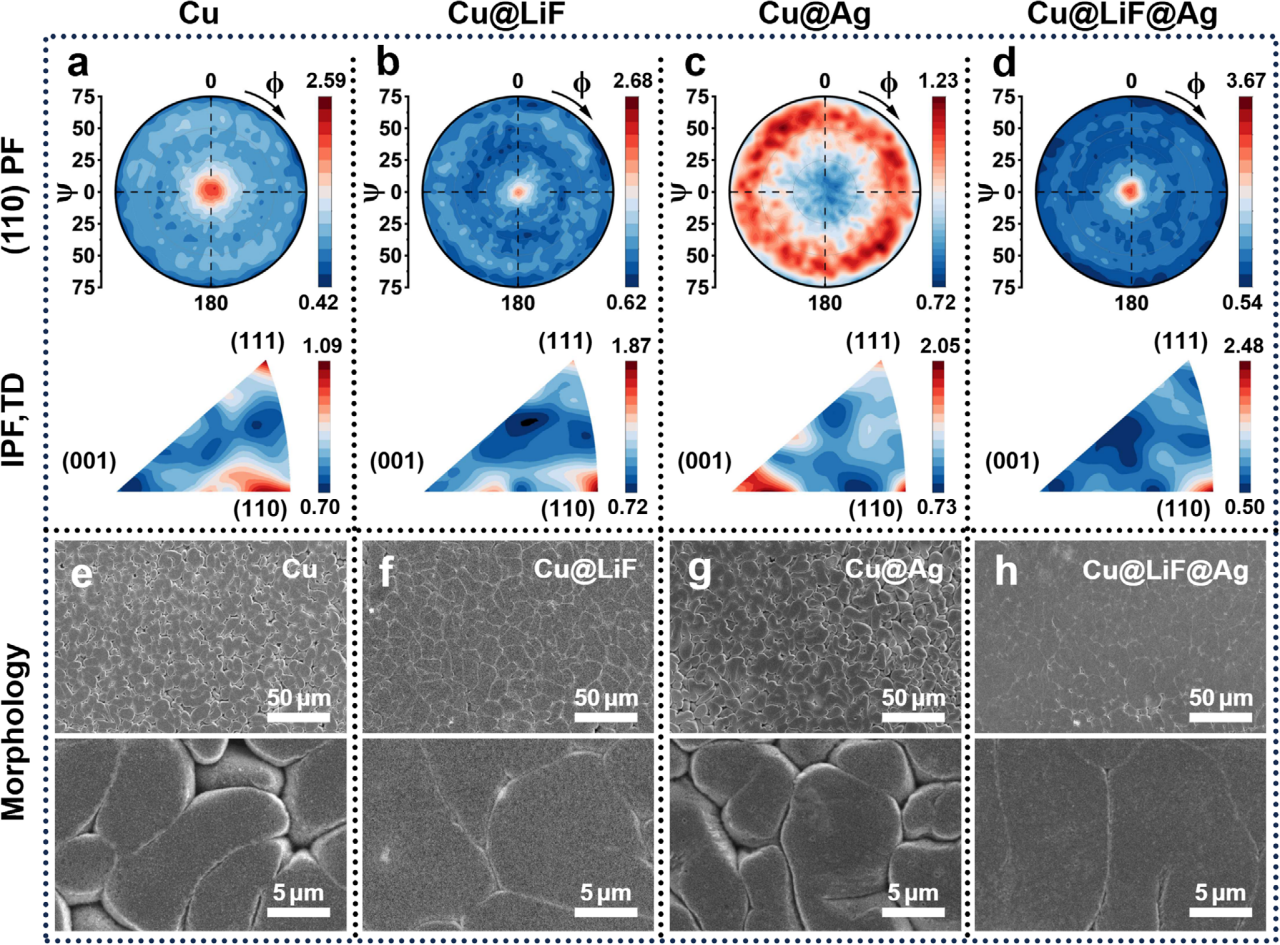
Textures and morphologies of the deposited Li on different substrates. Pole figures (PF) and inverse pole figures (IPF) obtained from XRD for 10 mAh cm^−2^ Li deposition on a) pristine Cu, b) Cu@LiF, c) Cu@Ag, and d) Cu@LiF@Ag. e–h) The corresponding SEM morphologies of the deposited Li.

**Figure** **2** and Figure S7 (Supporting Information) present the pole and inverse pole figures for Li deposited on the pristine Cu, Cu@LiF, Cu@Ag, and Cu@LiF@Ag substrates. Due to the combined influence of the {110}‐textured Cu substrate and the bath condition,^[^ ^6d^ ^]^ the (110) pole figure of Li deposited on pristine Cu (Figure 2a) shows a diffuse intensity distribution around the center, indicating a weak (110) preferred orientation. Compared with texture‐free electrolytic Cu (E‐Cu, Figure S8a, Supporting Information), the {110} texture of the rolled Cu substrate shows an epitaxial effect on the growth of Li, but the impact is limited owing to the large lattice mismatch. For the Li deposition layer on the Cu@LiF substrate, the (110) pole figure displays a distinct central spot with intensity converging into a ring at *ψ* = 60°, consistent with the rotational relationship of the {110} planes in the standard (110) projection.^[^ ^6a^ ^]^ This enhancement, along with the inverse pole figure (Figure 2b), confirms a strong {110} fiber texture with {110} planes parallel to the electrode surface. Conversely, the Cu@Ag substrate results in the loss of the {110} texture, evidenced by the absence of a central point in the (110) pole figure and a low intensity near the (110) corner in the inverse pole figure (Figure 2c). Most significantly, the Li deposited on Cu@LiF@Ag (Figure 2d) exhibits the highest central intensity in the (110) pole figure, and the inverse pole figure demonstrates a sharp (110) spot with over twice the intensity of the Li deposited on pristine Cu. These findings were further supported by the orientation distribution functions (ODFs), which effectively represent the key fiber texture components commonly found in BCC metals. The *φ*_2_ = 45° section of the ODF reveals a significantly stronger intensity near φ = 90° with a lower background for the Li deposited on Cu@LiF@Ag (Figure S9, Supporting Information). This feature confirms a strong Li {110} fiber texture. The lack of in‐plane orientation can be attributed to the absence of constraints normal to the growth direction. Therefore, the heteroepitaxial effect of the {110}‐textured Cu foil can be significantly enhanced by the LiF@Ag commensurate heterostructure, which acts as a buffer layer for Li growth.

In contrast, the preparation of a LiF@Ag modification layer on the E‐Cu substrate using the same deposition parameters does not induce the same enhancement in Li {110} texture. As shown in the (110) pole figure for the Li deposited on E‐Cu@LiF@Ag (Figure S8b, Supporting Information), a weak central spot is observed, and the intensity is broadly scattered. The corresponding inverse pole figure reveals weakly {111}‐textured Li on both the E‐Cu and E‐Cu@LiF@Ag substrates. This demonstrates that the epitaxial growth of Li on Cu@LiF@Ag inherits the texture of LiF and Ag that originates from the {110} texture of the rolled Cu substrate.

SEM imaging of the deposited Li correlates the observed crystallographic textures with deposition morphologies (Figure 2e–h). Li deposited on the pristine Cu exhibits gaps between irregular grains even at the high deposition capacity of 10 mAh cm^−2^ (Figure 2e). The Cu@LiF substrate promotes flatter deposition with larger and more regular grains showing an increased packing density (Figure 2f), likely due to the enhanced supercell matching between Li(110) and LiF(110). Obvious gaps between grains also exist on the Li deposited on Cu@Ag, and the grains become more disordered (Figure 2g), consistent with the loss of {110} texture (Figure 2c). Furthermore, the presence of small grains embedded between larger ones indicates a repeated Li nucleation behavior (Figure 2g).^[^ ^22^ ^]^ In stark contrast, Li deposited on Cu@LiF@Ag displays an ultra‐dense morphology with less prominent grain boundaries (Figure 2h). This can be attributed to the thermodynamically favored growth driven by reduced surface energy^[^ ^6d^ ^]^ and enhanced in‐plane Li⁺ diffusion kinetics that facilitate the filling of grain boundaries.^[^ ^7^, ^23^ ^]^

The electrocrystallization of Li encompasses nucleation, grain growth, and subsequent homoepitaxy, which are profoundly influenced by the substrate. Li adatoms generated from the reduction reaction in a solvated state diffuse to the electrode surface and nucleate on the substrate or previously formed grains. Early heterogeneous nucleation and substrate orientation are critical determinants of microstructure evolution and the formation of a compact film.^[^ ^24^ ^]^ Although Li nucleates unevenly on Cu@LiF due to the poor wettability of LiF (Figure S10, Supporting Information), the preferred orientation of the LiF layer strongly influences the crystallographic orientation of the Li nuclei. Subsequently, larger grains are formed due to the lateral expansion and grain coalescence. On Cu@Ag, the high lithiophilicity of Ag increases nucleation density, with characteristics of Li‐Ag alloying (Figure S10, Supporting Information). The concave flake‐like morphology suggests that Ag provides low‐barrier pathways for Li diffusion to the edges of growing nuclei.^[^ ^25^ ^]^ On the Cu@LiF@Ag substrate, the Ag NPs compensate for the poor wettability of LiF, promoting uniform nucleation with a higher density and earlier grain coalescence. Notably, the spherical Li nuclei on Cu@LiF@Ag, in stark contrast to the concave, flake‐like deposits on Cu@Ag, suggest that the LiF@Ag commensurate heterostructure suppresses the Li‐Ag alloying process.

Increasing the deposition capacity to an intermediate value of 5 mAh cm^−2^ (Figure S11, Supporting Information) leads to an increase in grain size across all samples. The grain size of Li on Cu@Ag is close to the final stage (10 mAh cm^−2^), confirming a multinucleation and multi‐layer growth manner. On Cu@LiF@Ag, the increased initial nucleation density driven by the Ag NPs results in slightly smaller grains compared to Cu@LiF. However, the Li grain size on Cu@LiF@Ag increases significantly from ≈6 µm to ≈13 µm as the deposition capacity increases from 5 to 10 mAh cm^−2^ (Figure S12, Supporting Information), indicating continuous growth without re‐nucleation and a high Li adatom diffusivity.^[^ ^23^ ^]^

### 2.3 Li Plating/Stripping on the Modified Substrates

A comparison analysis of pole figure results and the morphological evolution demonstrates the superior epitaxial capability of the Cu@LiF@Ag substrate. The LiF@Ag commensurate heterostructure effectively inherits the preferred orientation from the substrate, leading to a Li deposition layer with a pronounced {110} texture. Meanwhile, the distinct texture evolution on Cu@LiF and Cu@LiF@Ag indicates that substrate lattice mismatch is not the sole determinant of texture formation during nucleation and growth. Furthermore, the behavior of Ag differs fundamentally between the Cu@Ag and Cu@LiF@Ag substrates. Further electrochemical tests were performed to elucidate any difference in interfacial interactions during Li plating/stripping.

**Figure 3:**
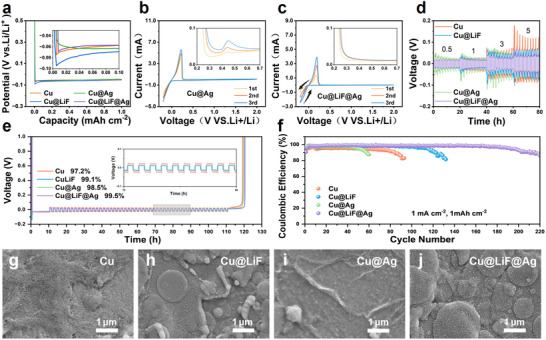
Electrochemical measurements of the modified substrates. a) Voltage profiles of galvanostatic Li deposition at 1 mA cm^−2^, with the inset showing the difference in nucleation overpotential. CV profiles of b) the Cu@Ag substrate and c) the Cu@LiF@Ag substrate recorded with a scan rate of 1 mV s^−1^. The insets show close‐ups of the anodic scan, where a distinct oxidation peak of Li dealloying is seen in (b) while being absent in (c). The black arrows in (c) indicate the nucleation loop of Li deposition. d) Rate performance of Li||Li symmetrical cells containing pristine Cu, Cu@LiF, Cu@Ag, and Cu@LiF@Ag pre‐deposited with 10 mAh cm^−2^ of Li, paired with 50 µm commercial Li foils. e) Average Coulombic efficiency (ACE) of different substrates with 10 mAh cm^−2^ of pre‐deposited Li cycled at 1 mA cm^−2^ and 1 mAh cm^−2^. f) Coulombic efficiency of Li||Cu cells containing different substrates. SEM morphology of g) the pristine Cu, h) Cu@LiF, i) Cu@Ag, and j) Cu@LiF@Ag substrates after a single Li plating/stripping cycle at 1 mA cm^−2^ and 1 mAh cm^−2^.

**Figure** **3**a shows the galvanostatic Li deposition profiles on the four different substrates at 1 mA cm^−2^ for 1 mAh cm^−2^. The Li affinity can be examined by analyzing the nucleation overpotential, which is defined as the difference between the bottom of the potential dip and the final plateau.^[^ ^25^ ^]^ After coating LiF on Cu, the Cu@LiF substrate exhibits a significantly elevated nucleation overpotential, consistent with the non‐uniform and localized Li nucleation (Figure S10, Supporting Information). This initial nucleation profoundly influences subsequent growth, delaying the coalescence of Li nuclei and consequently postponing the establishment of the voltage plateau. The Cu@Ag substrate demonstrates pronounced Li‐Ag alloying characteristics upon Li deposition, superseding the conventional nucleation process. This results in the elimination of the characteristic potential dip and a reduction of the overpotential to ≈0 V. In contrast, the formation of the LiF@Ag commensurate heterostructure on the Cu@LiF@Ag substrate induced a transition from the smooth potential slope associated with alloying (seen in Cu@Ag) to a distinct nucleation voltage dip. This signifies a shift in the dominant growth mechanism from alloying to nucleation‐driven growth. Nevertheless, compared to Cu@LiF, the Cu@LiF@Ag substrate displays significantly reduced nucleation and deposition overpotentials, indicating that the high lithiophilicity conferred by the Ag NPs persists even when the commensurate LiF layer suppresses the alloying pathway.

The electrochemical activity and reaction characteristics of the modified substrates were further investigated using cyclic voltammetry (CV). Li||Cu (pristine Cu, Cu@LiF, Cu@Ag, or Cu@LiF@Ag) half‐cells were assembled and measured at a scan rate of 1 mV s^−1^ between −0.2 and 2 V. The CV profiles (Figure 3b) show sharp reduction and oxidation peaks corresponding to Li plating and stripping. Consistent with the galvanostatic observations, the Cu@Ag substrate exhibits an overlap between the forward and backward scans below 0 V, characteristic of an alloying process. Furthermore, a distinct oxidation peak due to Li dealloying is visible upon magnification of the CV profiles (inset of Figure 3b).^[^ ^26^ ^]^ In contrast, both Cu@LiF (Figure S13, Supporting Information) and Cu@LiF@Ag (Figure 3c) display a higher cathodic current upon reversing the scan from −0.2 V, although the driving force for deposition decreases as the potential moves forward (hence, a nucleation loop is formed).^[^ ^27^ ^]^ This phenomenon occurs as Li deposition onto existing Li nuclei is kinetically more favorable than nucleation on the heterogeneous substrate, resulting in a higher reduction current at the same potential. Meanwhile, the disappearance of the dealloying peak on Cu@LiF@Ag is consistent with the galvanostatic Li stripping profiles (Figure S14, Supporting Information), where the final dealloying potential plateau on Cu@Ag is absent on Cu@LiF@Ag. The commensurate LiF layer alters the behavior of Ag NPs, transforming the early Li deposition from alloying‐governed to a nucleation‐driven growth process.

The rate performance of Li||Li symmetrical cells, pre‐deposited with 10 mAh cm^−2^ of Li, was evaluated across current densities from 0.5 to 5 mA cm^−2^ (Figure 3d). While all cells exhibit higher overpotentials with increasing current density, cells using the Cu@LiF@Ag and Cu@LiF substrates consistently show reduced increments in overpotential. This improved performance is consistent with the observed enhancements of the {110} texture of the deposited Li. The Cu@Ag cell, however, exhibits considerable polarization, particularly at 3 mA cm^−2^, stemming from the energy barriers inherent to the Li‐Ag alloying/dealloying process.^[^ ^28^ ^]^ Notably, the Cu@LiF@Ag cell demonstrates superior high‐rate capability, maintaining a remarkably low overpotential of ≈50 mV at 5 mA cm^−2^. This enhanced performance is attributed to the combination of uniform Li deposition morphology and the intrinsically faster Li^+^/Li redox kinetics associated with the Li(110) plane.^[^ ^6d^ ^]^

Li plating/stripping reversibility was quantified by measuring the ACE^[^ ^29^ ^]^ over 50 cycles in cells pre‐deposited with 10 mAh cm^−2^ of Li. As shown in Figure 3e, Li deposited on the pristine Cu substrate yields a low ACE of 97.2%. Although the lithiophilic Ag in Cu@Ag improves the reversibility of plating/stripping, the intermetallic compounds (e.g., LiAg_3_) formed as a result of Li‐Ag alloying are resistant to stripping,^[^ ^14b^ ^]^ limiting further enhancement of the ACE (slightly increased to 98.1%). In stark contrast, Li deposited on the Cu@LiF@Ag substrate achieves a significantly improved ACE of 99.5%, higher than the value obtained on the Cu@LiF substrate (99.1%). This demonstrates that the epitaxial growth of {110}‐textured Li, facilitated by both the Cu@LiF@Ag and Cu@LiF substrates, substantially enhances the reversibility of the Li plating/stripping process.

Long‐term cycling stability was assessed by monitoring the CE of Li||Cu cells cycled at 1 mA cm^−2^ with a capacity of 1 mAh cm^−2^ and a stripping cutoff at 1.0 V (Figure 3f). The CE of the cell containing the pristine Cu substrate decreases rapidly from 95.3% after 80 cycles. The Cu@Ag cell exhibits an improved initial CE (97.5%), but suffers from limited stability. The CE falls rapidly after only 50 cycles, likely due to the degradation of the Ag structure during the repeated alloying and dealloying process. The Cu@LiF cell demonstrates improved stability, reaching ≈120 cycles with a CE of ≈98.2% before gradually decaying. Most impressively, the Cu@LiF@Ag cell maintains the highest CE of ≈99.1% among the four cells for over 160 cycles, signifying its superior cycling stability and more than doubled cycle life compared to the pristine Cu.

Post‐cycling morphological analysis provides direct evidence that correlates the observed electrochemical performance with substrate integrity and deposition characteristics. After a single plating/stripping cycle, the pristine Cu substrate exhibits a significant accumulation of inactive species, including SEI components and “dead Li” (Figure 3g). This can be attributed to the inherent non‐uniform deposition on lithiophobic Cu, accompanied by continuous parasitic reactions.^[^ ^30^ ^]^ Conversely, the Cu@LiF substrate presents a relatively clean post‐stripping surface (Figure 3h), consistent with its enhanced CE. The reduced parasitic reactions result from the chemical inertness of LiF and a more efficient stripping process enabled by the improved kinetics of the {110}‐textured Li deposition layer.^[^ ^6i^ ^]^ In the case of Cu@Ag, while Ag is known to enhance the lithiophilicity via alloying, repeated cycling leads to degradation or blocking of the lithiophilic sites,^[^ ^11^ ^]^ leading to the formation of “dead Ag” and the accumulation of inert phases like LiAg_3_.^[^ ^11^ ^]^ This is confirmed by Figure 3i, which reveals the loss of structural integrity for the Ag particles. The latter increases the surface area exposed to the electrolyte, accelerating parasitic reactions and shortening cell lifespan.^[^ ^11^, ^12^ ^]^

Remarkably, the Cu@LiF@Ag substrate displays a clean surface after Li stripping and retains the intact Ag NPs morphology (Figure 3j), similar to the as‐prepared state (Figure 1e). The integrity of the LiF@Ag heterostructure can even be retained up to 40 cycles of Li plating/stripping in the Li||Cu cell (Figure S15, Supporting Information). Besides the progressive formation of an SEI layer, the original morphology of the Ag NPs and LiF flakes remains clearly visible, indicating that the LiF@Ag heterostructure does not undergo significant degradation over extended cycles. This observation supports the conclusion that Li deposition occurs via epitaxial growth without Ag alloying, enabling the Ag NPs to durably guide the formation of {110}‐textured Li over repeated cycles, thereby delivering enhanced CE and prolonged cycle life.

### 2.4 Li Heteroepitaxial Growth on the LiF@Ag Commensurate Heterostructure

The electrochemical measurements identify two distinct Li deposition mechanisms: an alloying‐dominated process on Cu@Ag and a nucleation‐driven epitaxial growth process on Cu@LiF and Cu@LiF@Ag. Notably, the non‐alloying Ag NPs on LiF preserve its lithiophilicity and significantly facilitate the epitaxial growth pattern, promoting a dense and uniform Li deposition layer with a strong {110} texture. While epitaxial growth typically requires crystallographic compatibility, the observed epitaxy between BCC Li and the rock salt LiF and FCC Ag requires further investigations to elucidate the specific interfacial interactions that enable the heteroepitaxial growth, including lattice mismatch across relevant crystallographic planes and the synergistic effect of the LiF@Ag commensurate heterostructure that governs the non‐alloying nature of Ag NPs on LiF, which is pivotal to the observed enhancements in electrochemical performance.

**Figure 4:**
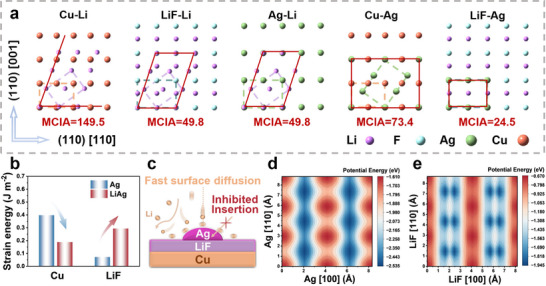
Interfacial properties of the modified substrates. a) The (110) plane lattice mismatches of Cu, LiF, and Ag against Li, as measured by the minimal coincident interface area (MCIA) marked by the red lines. The areas surrounded by the dotted lines are the (110) cross‐section of the unit cells. The large MCIA value characterizes the large (110) plane lattice mismatch between Ag and Cu compared to the commensurate interface between Ag and LiF. b) The change of strain energy for the transition from Ag to the β phase (LiAg) on Cu(110) and LiF(110). c) A schematic illustration of the inhibited Li insertion for Li‐Ag alloying and the fast surface diffusion of Li adatoms on the LiF@Ag commensurate heterostructure. The potential energy surface of Li adatoms on d) Ag(110) and e) LiF(110).

The quantitative assessment of lattice mismatch at heterointerfaces is crucial for understanding and predicting epitaxial growth phenomena. Methods relying solely on the percentage difference in lattice parameters are inadequate when the interfacing materials possess distinct crystal symmetries. A more comprehensive method, the minimal coincident interface area (MCIA),^[^ ^8^, ^31^ ^]^ addresses this limitation by considering the relative crystallographic orientations and the potential for commensurate matching over extended interfacial domains via coincident site lattices. Applying MCIA to the deposition of Li on the pristine Cu substrate reveals significant crystallographic incompatibility, characterized by a large MCIA value of 149.5 (**Figure** **4**a). This indicates that Cu is not an ideal substrate for epitaxial growth, leading to poorly aligned deposition.

In contrast, introducing Ag and LiF significantly enhances lattice compatibility, reducing the MCIA to a more favorable value of 49.8. Although initial nucleation on the Cu@LiF substrate exhibits spatial inhomogeneity, the significantly reduced lattice mismatch promotes the alignment of Li grains with the substrate's preferred orientation from the nucleation stage. Subsequent growth involves the coalescence of adjacent grains, promoting the (110)‐oriented heteroepitaxial growth.

Depending on the underlying layer, the modified substrates containing Ag exhibit fundamentally distinct characteristics in Li deposition. While both Cu@Ag and Cu@LiF@Ag possess the low‐lattice‐mismatch Ag component, Li deposition on Cu@Ag results in randomly oriented deposits. Conversely, the Cu@LiF@Ag substrate facilitates the growth of a {110}‐textured Li layer. This critical difference arises from the distinct interfacial reactions between the deposited Li and the Cu@Ag and Cu@LiF@Ag substrates. As shown in Figure 4a, the lattice mismatch between Ag(110) and Cu(110) is relatively large (MCIA = 73.4), indicating limited crystallographic constraint imposed by the Cu substrate on Ag, particularly during the Li‐Ag alloying processes. At the initial stage of Li deposition, Li dissolves into Ag to form the *α* phase (solid solution), and the orientation of Ag starts to shift in response to the lattice distortion.^[^ ^14^, ^32^ ^]^ However, as Li concentration increases beyond the α‐phase solubility limit (approaching a Li: Ag atomic ratio of 0.3), a phase transition to the *β* phase (LiAg intermetallic) will occur, allowing for further Li incorporation.^[^ ^33^ ^]^ Density functional theory (DFT) calculations indicate that the MCIA between the *β* phase and Cu will further decrease (Figure S16, Supporting Information), facilitating phase transformation from Ag to the *β* phase on Cu and completely disrupting the epitaxial growth. Furthermore, the significant negative binding energy (−2.85 J m^−2^) and low interface energy (0.67 J m^−2^) calculated for the interaction between Cu and the β phase (**Table** **1**; Figure S17, Supporting Information) suggest that this transformation from Ag to the β phase is thermodynamically favored on a pristine Cu substrate.

**Table 1:** The binding energy, interface energy, and strain energy of different interfaces.

Interface	Binding energy [J m^−2^]	Interface energy [J m^−2^]	Strain energy [J m^−2^]
Cu(110)/Ag(110)	−2.50	1.35	0.41
Cu(110)/LiAg(110)	−2.85	0.67	0.19
LiF(110)/Ag(110)	−0.88	1.81	0.07
LiF(110)/LiAg(110)	−0.88	1.45	0.30

In contrast, the commensurate interface of Ag and LiF induces a composition pinning effect, where the lattice of the Ag phase tends to be ‘pinned’ at a value that is lattice‐matched to the LiF substrate, even though it could vary significantly under unconstrained conditions.^[^ ^15^, ^34^ ^]^ This phenomenon arises from the strict epitaxial relationship between Ag and LiF, underpinned by their structural similarities (FCC vs. rock salt) and closely matched lattice parameters (Ag: 4.10 Å; LiF: 4.08 Å), resulting in a very low MCIA of 24.5 (Figure 4a). This effect is further enhanced by the lattice contraction of Ag NPs, which occurs due to surface energy minimization.^[^ ^35^ ^]^ DFT calculations indicate that the phase transition of Ag to the *β* phase on a LiF substrate will induce a positive change in the binding energy (Table 1), making the Li‐Ag alloying process thermodynamically unfavorable.

From a kinetic perspective, the electrochemical insertion of Li into Ag NPs is a diffusion‐driven process. Interfacial phenomena, such as lattice strain and altered diffusion kinetics, dominate the Li‐Ag alloying process at the microscopic scale. Replacing the pre‐existing LiF and Ag interface with a new interface between LiF and the *β* phase presents a substantial energy barrier during initial growth. The sharp increase in strain energy (Δ*E*_strain energy_ = 0.22 J m^−2^, Figure 4b) becomes the primary impediment to Li alloying for the Ag NPs. Consequently, fast surface diffusion of Li adatoms on Ag replaces bulk diffusion, resulting in an inhibited insertion (Figure 4c). Hence, the LiF@Ag commensurate heterostructure prevents the Li‐Ag alloying process, ensuring the structural integrity of the Ag NPs along repeated charge and discharge. The surface characteristics of supercell matching and facilitated diffusion are retained to promote Li heteroepitaxial growth.

The transient electrochemical dynamics of Li deposition processes cause the system to deviate from equilibrium thermodynamics, resulting in metastable states dominated by surface diffusion of adsorbed adatoms. DFT‐calculated potential energy surfaces reveal that both Cu(110) and Ag(110) exhibit higher adsorption energies for Li adatoms compared to LiF(110) (Figure 4d–e; Figure S18, Supporting Information). The stable binding sites for adsorbed Li adatoms are located at the surface hollow sites on Cu(110) and Ag(110), with the transition state for the adsorbed adatoms positioned at the bridge sites (Figure S19, Supporting Information). Both metals display relatively low diffusion barriers (Cu: ≈0.14 eV; Ag: ≈0.13 eV). Classical nucleation theory suggests that higher adsorption energy should correlate with reduced nucleation overpotential.^[^ ^32^ ^]^ However, neither metal fully exploits this surface feature for ideal Li deposition, with Cu being hampered by its high lattice mismatch with Li and Ag undergoing alloying with Li.

The less prominent {110}‐textured Li deposition on the Cu@LiF substrate can be attributed to the restricted interfacial dynamics of LiF. Although LiF adopts a rock salt structure similar to FCC, it presents a disparate potential energy surface due to the combined influence of surface and subsurface F ions. This interaction bifurcates the stable adsorption site and positions the transition state centrally at the midpoint between them (Figure 4e; Figure S19, Supporting Information), resulting in a comparatively higher diffusion barrier (0.37 eV) that confines the (110)‐oriented heteroepitaxy to be dominated by thermodynamically driven lattice matching. However, on the Cu@LiF@Ag substrate, the limitations of both LiF and Ag are addressed by modulating the interface dynamics through the use of the LiF@Ag commensurate heterostructure. DFT calculations suggest a low strain energy (0.0718 J m^−2^, Table 1) of the interface between LiF and Ag, which means that Ag can retain its inherent high lithiophilicity and low adatom diffusion barrier. This significantly reduces the relatively high nucleation overpotential of LiF and cooperates with the proper lattice matching to achieve the ultra‐dense and highly {110}‐textured Li deposition on the Cu@LiF@Ag substrate.

### 2.5 SEI Formation and Full Cell Performance

**Figure 5:**
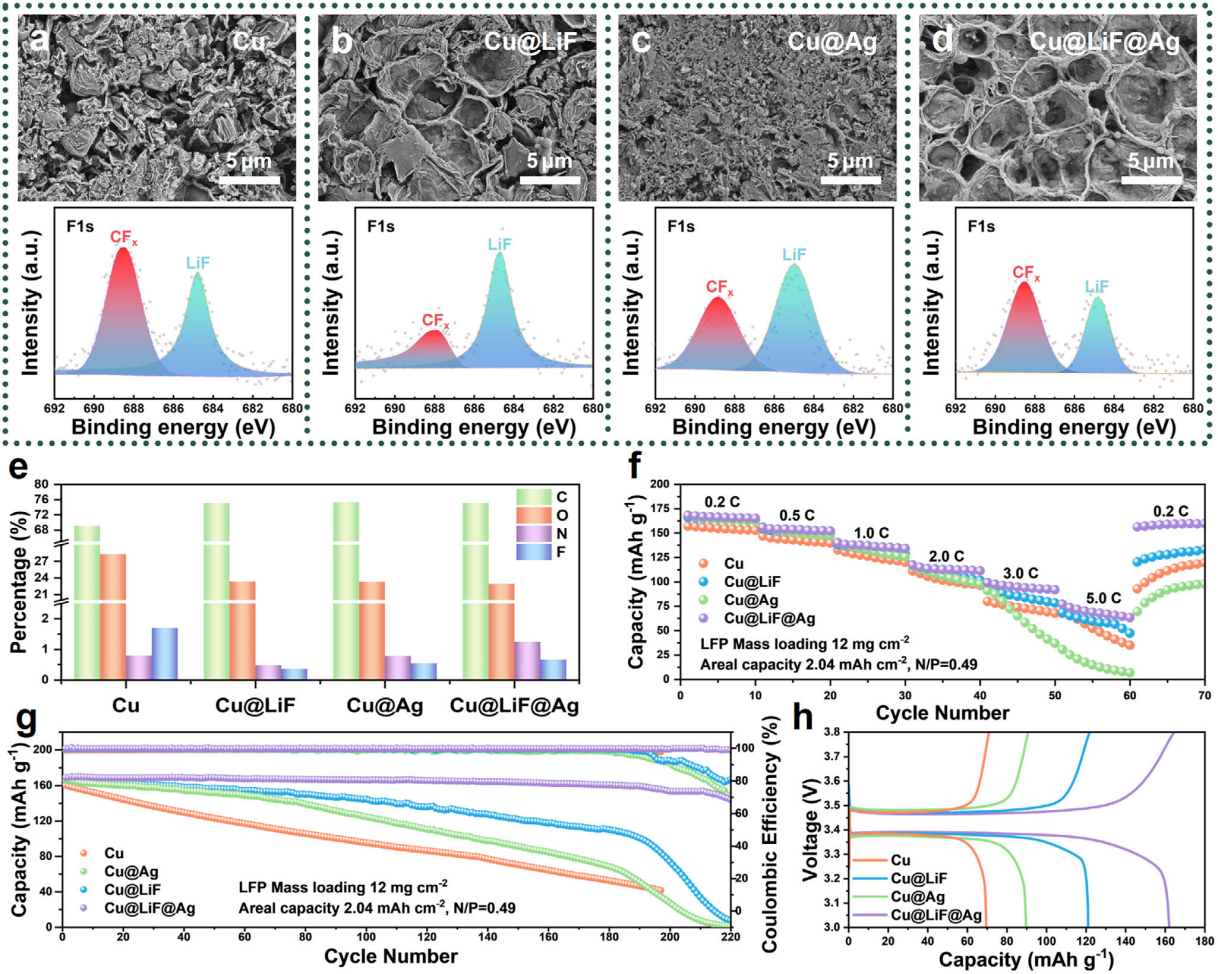
SEI formation and full cell performance. SEM morphologies and XPS F1s spectra of the SEI formed on a) the pristine Cu, b) Cu@LiF, c) Cu@Ag, and d) Cu@LiF@Ag substrates after 15 cycles of Li plating and stripping at 1 mA cm^−2^ and 1 mAh cm^−2^. e) The elemental compositions of C, O, N, and F for the SEI formed on the four substrates. f) High‐rate and g) charge/discharge cycling performance of LiFePO_4_ full cells. h) Charge and discharge profiles at cycle 150 taken from the cycling tests shown in (g). The areal capacity of the cathode is 2.04 mAh cm^−2^. Substrates with 1 mAh cm^−2^ pre‐deposited Li were used as the anodes, leading to the low N/P ratio of 0.49.

The formation and characteristics of the SEI layer are crucial for the performance and stability of Li metal batteries. SEM analysis reveals distinct SEI morphologies after 15 Li plating/stripping cycles. The pristine Cu substrate exhibits a thick and heterogeneous SEI structure (**Figure** **5**a), whereas the Cu@LiF substrate promotes a hollow SEI morphology (Figure 5b) due to the epitaxial growth of large Li grains primarily exposing the (110) facet. In contrast, the SEI on Cu@Ag is dominated by fine particles (Figure 5c), indicative of an unstable interface with “dead Li” formation resulting from Li‐Ag alloying and parasitic reactions between the randomly oriented Li deposits and the electrolyte.

The Cu@LiF@Ag substrate promotes the formation of a smooth, well‐defined SEI skeleton that mainly develops at grain boundaries (Figure 5d). This morphology arises from a bottom‐up epitaxial growth process, which minimizes parasitic reactions by exposing the relatively stable Li(110) plane to the electrolyte. The resultant wrinkle‐free and interconnected SEI structure effectively isolates active sites, enhances Li^+^ diffusion across grain boundaries, and promotes the reversible plating/stripping dynamics inherent to the Li(110) plane.^[^ ^36^ ^]^

X‐ray photoelectron spectroscopy (XPS) provides insights into the compositional variations of the SEI. While carbon species, likely from the oxidative oligomerization of the electrolyte solvent,^[^ ^6d^ ^]^ dominate the SEI on all substrates, notable differences emerge in F and N contents (Figure 5e; Figure S20, Supporting Information). On pristine Cu, extensive parasitic reactions lead to a high F content, dominated by the products of incompletely decomposed Li bis (trifluoromethanesulfonyl) imide (LiTFSI) salt (CF_x_) rather than LiF (Figure 5a). Conversely, Cu@LiF shows significantly reduced F content but a markedly higher proportion of LiF (Figure 5b), suggesting that (110)‐oriented epitaxial growth suppresses overall salt decomposition yet favors the complete decomposition of LiTFSI to LiF.^[^ ^37^ ^]^ The Cu@Ag substrate exhibits elevated CF_x_ levels (Figure 5c), consistent with the accelerated electrolyte consumption by the randomly oriented Li deposits, hindering the complete C─F bond cleavage required for LiF formation.^[^ ^38^ ^]^ On Cu@LiF@Ag, although the CF_x_ peak intensity is slightly higher than LiF (Figure 5d), the overall F content and LiF‐to‐CF_x_ ratio suggest a relatively stable LiF proportion within the SEI (Figure 5e), indicating that Ag NPs facilitate CF_x_ formation without significantly diminishing LiF production. Meanwhile, the enhanced organic components improve the interface elasticity, resulting in a robust SEI skeleton.

Analysis of the N1s spectra (Figure S21, Supporting Information) exhibits a similar salt decomposition manner influenced by the {110}‐textured Li deposits. On the Cu@LiF and Cu@LiF@Ag substrates, an increased proportion of Li_3_N is formed via the complete reduction of the LiNO_3_ additive.^[^ ^39^ ^]^ While the pristine Cu surface shows significant amounts of intermediates formed by the incomplete reduction of LiNO_3_ (LiN_x_O_y_), they are completely reduced into Li_3_N on Cu@LiF. On the Cu@Ag substrate, Ag appears to favor incomplete LiNO_3_ reduction, resulting in a sharp increase of LiN_x_O_y_ in the SEI. The Cu@LiF@Ag substrate exhibits the highest N and Li_3_N content (Figure 5e; Figure S21, Supporting Information) due to a synergistic effect, where Ag facilitates initial LiNO_3_ decomposition, and the facet exposure at grain boundaries caused by the {110}‐dominated deposition provides favorable energetic conditions for a complete reduction to Li_3_N.^[^ ^40^ ^]^ Electrochemical impedance spectroscopy (EIS) measurements in the stripped state after 15 cycles suggest that the SEI impedance for all modified substrates is significantly lower than that of the pristine Cu substrate (Figure S22, Supporting Information). The lowest SEI impedance of the Cu@LiF@Ag substrate directly correlates with the higher content of ionically conductive species (LiF and Li_3_N)^[^ ^13b^ ^]^ observed in the XPS analysis.

Full cells with a low N/P ratio of 0.49 were assembled to evaluate the practical applicability of different substrates. At the high‐rate regime, the lithiophilic benefits of Ag are entirely lost by the detrimental impact of Li‐Ag alloying, causing the Cu@Ag cell to exhibit the poorest high‐rate capability (Figure 5f). Conversely, substrates that promote {110}‐textured Li (Cu@LiF and Cu@LiF@Ag) demonstrate significantly improved high‐rate performance (70 mAh cm^−2^ for Cu@LiF and 75 mAh cm^−2^ for Cu@LiF@Ag at 5 C). Upon returning to a lower rate (0.2 C), the Cu@LiF@Ag cell displays remarkable reversibility (159.6 mAh cm^−2^), recovering 94.9% of its initial capacity and substantially outperforming Cu@LiF (130 mAh cm^−2^, 81.7%), Cu (119.6 mAh cm^−2^, 61.2%), and Cu@Ag (97.8 mAh cm^−2^, 56.8%). The synergistic effect of LiF and Ag in Cu@LiF@Ag not only overcomes the limitations of Li‐Ag alloying at high rates but also provides a robust and stable (110)‐oriented epitaxial substrate that significantly outperforms both Cu@Ag and bare Cu across a wide range of current densities. Voltage profiles (Figure S23, Supporting Information) further confirm the accelerated degradation of the Cu@Ag anode at high rates, evidenced by a pronounced suppression of the voltage plateau as the charge and discharge rates increase. Further tests were conducted using LFP cathodes with a higher mass loading of 26 mg cm^−2^, achieving a commercially relevant areal capacity of 4.42 mAh cm^−2^. The Cu@LiF@Ag cell also demonstrates the best high‐rate performance and capacity retention up to 5 C and an elevated temperature of 40 °C (Figure S24, Supporting Information).

Long‐term cycling tests were then performed at 0.5 C (Figure 5g), which further confirms the significantly improved cycle life of the Cu@LiF@Ag anode at the low N/P ratio. The Cu@Ag cell exhibits slightly improved capacity retention compared to the pristine Cu cell before 190 cycles. This suggests that the low‐content Ag on the Cu substrate benefits the initial cycling stability at low current densities owing to its high lipophilicity. After 150 cycles, despite all four cells demonstrating relatively stable operating voltage profiles, the Cu@Ag cell exhibits the most pronounced polarization during the charge and discharge processes (Figure 5h). In stark contrast, the Cu@LiF and Cu@LiF@Ag cells exhibit significantly improved cycling performance due to the stable {110}‐textured Li deposition. The Cu@LiF cell delivers a higher specific capacity of 100.7 mAh cm^−2^ after 190 charge and discharge cycles compared with Cu and Cu@Ag (46.5 and 51.3 mAh cm^−2^). The Cu@LiF@Ag cell presents the best long‐term stability, with a specific capacity of 143.9 mAh cm^−2^ and a retention of 85.1% after 220 cycles. Compared with recent works on modified Cu substrates for Li metal anodes (Table S2, Supporting Information), the achieved ACE of the Cu@LiF@Ag substrate is among the highest, leading to relatively longer full cell cycle life under a lower N/P ratio.

## 3 Conclusion

In conclusion, a LiF@Ag commensurate heterostructure is developed that combines interfacial kinetic modulation with the conventional lattice matching of heteroepitaxy to achieve {110}‐textured Li metal anodes. The composition pinning effect of the LiF@Ag commensurate heterostructure effectively avoids the Li‐Ag alloying process and ensures the structural stability of LiF@Ag in repeated charge and discharge. DFT calculations further reveal that stabilization of the Ag phase in the LiF@Ag commensurate heterostructure originates from a sharp increase in strain energy that hinders the Li‐Ag alloying process on LiF, while the same process on Cu is thermodynamically favorable. Meanwhile, Ag contributes to a significantly reduced Li adatom diffusion barrier compared with LiF, which improves interfacial dynamics to enhance the heteroepitaxial effect on top of the high lattice matching between Li and LiF. Consequently, the Cu@LiF@Ag substrate enables ultra‐dense and dendrite‐free Li heteroepitaxial deposition with prominent {110} texture. The resulting {110}‐textured Li metal anodes exhibit significantly improved electrochemical performance, including higher CE, prolonged cycle life, and enhanced high‐rate capability even in low N/P ratio full cells. The strategy to construct commensurate heterostructures can guide future material screening for designing heteroepitaxy substrates toward textured LMAs with enhanced efficiency and interfacial stability.

## Conflict of Interest

The authors declare no conflict of interest.

## Supporting information



Supporting Information

## Data Availability

The data that support the findings of this study are available from the corresponding author upon reasonable request.
