# Supplementary material for: Durable Lithium Metal Anodes Enabled by {110}‐Textured Epitaxy on a LiF@Ag Commensurate Heterostructure
Source: Adv Sci (Weinh). 2025 Aug 7;12(41):e10886. doi: 10.1002/advs.202510886 (PMC12591140; doi:10.1002/advs.202510886)
Supplement: Supplementary file 1 — Supporting Information [file ADVS-12-e10886-s001.docx]

Supporting Information

**Durable Lithium Metal Anodes Enabled by {110}-textured Epitaxy on a LiF@Ag Commensurate Heterostructure**

*Liming Zhang, Xuemin Wang, Xiaotong Sun, Haobo Yang, Xinyi Miao, Zifeng Gu, Chuanzhong Chen* and Cheng Hu**

Experimental Section

*Preparation of the modified Cu substrates*: LiF and Ag were deposited on the rolled Cu substrates by PLD using a neodymium-doped yttrium aluminum garnet (Nd:YAG) 532 nm solid-state laser source (BEAMTECH, SGR 30). The Cu substrates were cleaned in 1 M H2SO4 to remove the surface oxidation layer, washed with deionized water and anhydrous ethanol, and then vacuum-dried at 25 °C. The deposition was carried out in a vacuum condition of 5 × 10^−4^ Pa with a laser flux of 3 J cm^-2^, 25 ℃ and a repetition rate of 10 Hz. The target-to-substrate distance was kept at 35 mm. A cylindrical LiF target with a diameter of 20 mm and a thickness of 5 mm was made of LiF powder (ALADDIN, 99.99%) by pressing at a pressure of 325 MPa and sintering at 300 ℃ for 2 h. The Ag target was commercial (ZHONGKE YANNUO, 99.99%) with a diameter of 20 mm and a thickness of 5 mm. Cu@LiF and Cu@Ag were obtained by depositing with the LiF and Ag targets for 5 and 10 min, respectively. Cu@LiF@Ag was prepared by depositing LiF and Ag sequentially for the same periods.

*Electrochemical measurements:* CR2032 coin cells were assembled in an Ar-filled glovebox with water and oxygen concentrations lower than 0.1 ppm. Li||Cu half-cells were assembled using the pristine rolled Cu, Cu@LiF, Cu@Ag, or Cu@LiF@Ag as the working electrodes, 50 μm thick Li foils as the counter and reference electrodes, and CELGARD 2325 porous membranes as the separator. The electrolyte was 1 M LiTFSI dissolved in 1,3-dioxolane (DOL)/dimethyl ether (DME) (v/v=1:1) containing 2% LiNO_3_. Excessive electrolyte (30 µL on each side of the cell) was used to completely wet the electrodes and separator. For the LFP full cells, the cathode LFP loading was 12 mg cm^-2^ with a theoretical areal capacity of 2.04 mAh cm^-2^, and the anodes were prepared by pre-depositing 1 mAh cm^-2^ of Li on the four different substrates. This configuration delivers a low N/P ratio of 0.49. The same ether-based electrolyte was employed to assemble the full cells. Galvanostatic Li deposition was conducted on the four substrates at 1 mA cm^-2^ with a capacity limit of 1 mAh cm^-2^. CV was measured between -0.2 and 2 V at a scan rate of 1 mV s^-1^. The ACE was determined by pre-plating 10 mAh cm^-2^ of Li at 1 mA cm^-2^, followed by 50 cycles of Li plating/stripping (1 mA cm^-2^, 1 mAh cm^-2^), and a final complete Li stripping to quantify the irreversible capacity loss. CE of Li||Cu cells was measured at 1 mA cm^-2^, 1 mAh cm^-2^ with a 1.0 V stripping cutoff. Rate capability was assessed in Li||Li symmetric cells (10 mAh cm^-2^ pre-deposited Li) under current densities spanning from 0.5 to 5 mA cm^-2^. EIS was performed from 0.01 Hz to 100 kHz with an AC amplitude of 5 mV, using Li||Cu half-cells after 15 cycles of plating/stripping.

*Materials characterization:* Surface morphologies of the substrates, the deposited Li and the SEI were characterized using SEM (ZEISS, Gemini 500) operated at 5.0 kV. The elemental composition and chemical state analysis of the SEI were performed by using XPS (THERMO SCIENTIFIC, K-Alpha) with an Al *k*_α_ source. XRD and pole figure analysis were performed on a RIGAKU Smartlab diffractometer equipped with a Cu *k*_α_ source. Diffraction patterns were obtained using the Bragg-Brentano optics with a focused beam. The RTC of different Li planes were determined according to:

$I will schedule some time for us to connect.\text{RTC}_{\left( hkl \right)}=\frac{I_{\left( hkl \right)}/I_{\left( hkl \right)}^{0}}{\sum_{1}^{n} I_{\left( hkl \right)}/I_{\left( hkl \right)}^{0}}\times100\%$

Pole figure analysis was conducted using a parallel beam geometry with a 2 mm incident beam slit. Li deposited specimens were covered by multiple layers of Kapton tape in the Ar-filled glovebox to avoid damage by the air during measurements. Pole figures were collected in the *φ* range of 0 to 360° and *ψ* range of 0 to 75° at a step size of 5°. Inverse pole figures and ODFs were calculated using the SmartLab Studio Ⅱ software.

*Theoretical calculations:* DFT calculations were performed using the CP2K package with the PBE functional and the DZVP-MOLOPT-GTH basis sets.^[1]^ Grimme’s DFT-D3(BJ) correction was applied to better describe dispersion interactions.^[2]^ Calculations were performed using the XY periodicity with the Martyna-Tuckerman formalism,^[3]^ 3×3×1 k-point grids, and Fermi-Dirac smearing at 300 K. A vacuum layer with the thickness that equals the z-span of atomic coordinates plus an extra 10 Å was added to the slab models. Interface models were built with 5-layer slabs on both sides of the interface. The size of the slabs was determined by the MCIA as shown in Figure S17. Two layers near the interface were relaxed to the RMS force threshold of 3×10^−4^. The potential energy surfaces of Li adatom adsorption were calculated using 3-layer (3×3) slabs of Cu(110), Ag(100) and LiF(110). The Li adatom was placed at 25 symmetric positions above a unit cell of the slab at a Z separation of 3.0 Å and then relaxed in Z together with the first layer of the slab. Surface energy was calculated *via*:

$\gamma_{\text{surface}\text{ energy}}=\frac{E_{\text{slab}}-N\cdot E_{\text{bulk}}}{2A}$ (1)

where *E*_slab_ is the total energy of the slab, *N* is the number of atoms in the unit cell, and *E*_bulk_ is the single-atom energy of the bulk unit cell. The binding energy of slab A and B is calculated using:

$E_{\text{bind}\text{ing energy}}=\frac{E_{\text{com}}-\left( E_{\text{slabA}}+E_{\text{slabB}} \right)}{A}$ (2)

where *E*com is the total energy of the combined system, *E*_slabA_ and *E*_slabB_ are the energies of the isolated slabs, and *A* is the interface area. The interface energy is the sum of the binding energy and the surface energy of the isolated slabs:

$\gamma_{\text{interface energy}}=\frac{E_{\text{combined}}-\left( E_{\text{slabA}}+E_{\text{slabB}} \right)}{A}+\left( \gamma_{\text{A}}+\gamma_{\text{B}} \right)$ (3)

and the strain energy is the energy difference of the combined interface model of slab A and B before and after relaxation:

$E_{\text{strain energy}}=E_{\text{com}}^{\text{unrelaxed}}-E_{\text{com}}^{\text{relaxed}}$ (4)

**Supplementary Figures**


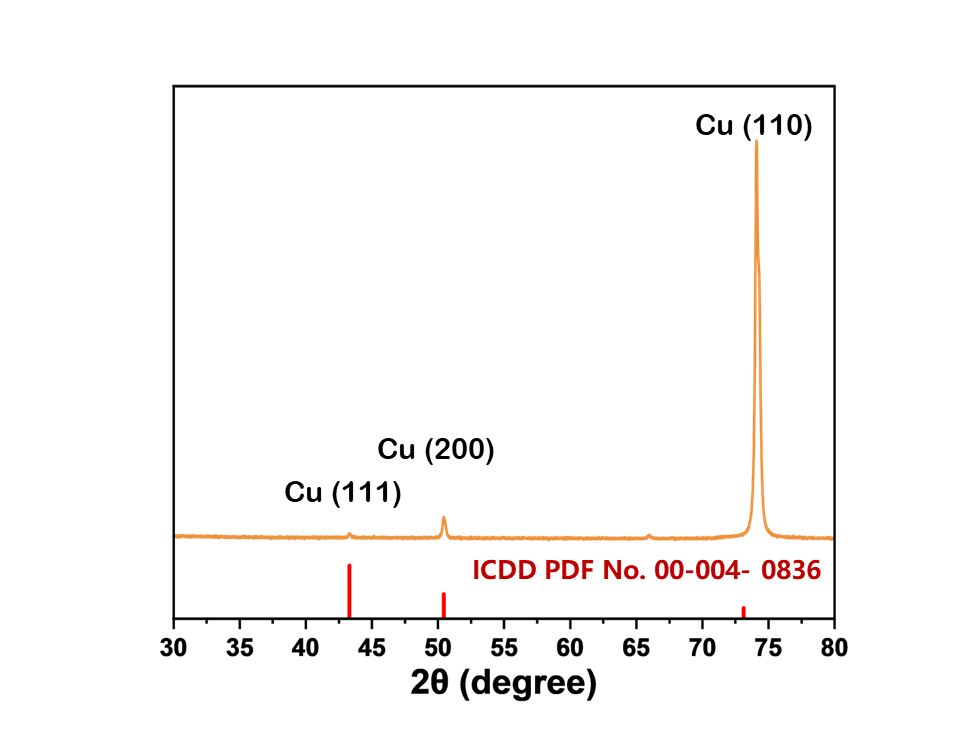


**Figure S1.** XRD pattern of the pristine rolled Cu foil used as the pristine Li deposition substrate. The dominating intensity of the (110) reflection indicates that the pristine Cu foil is {110}-textured as a result of the rolling process.


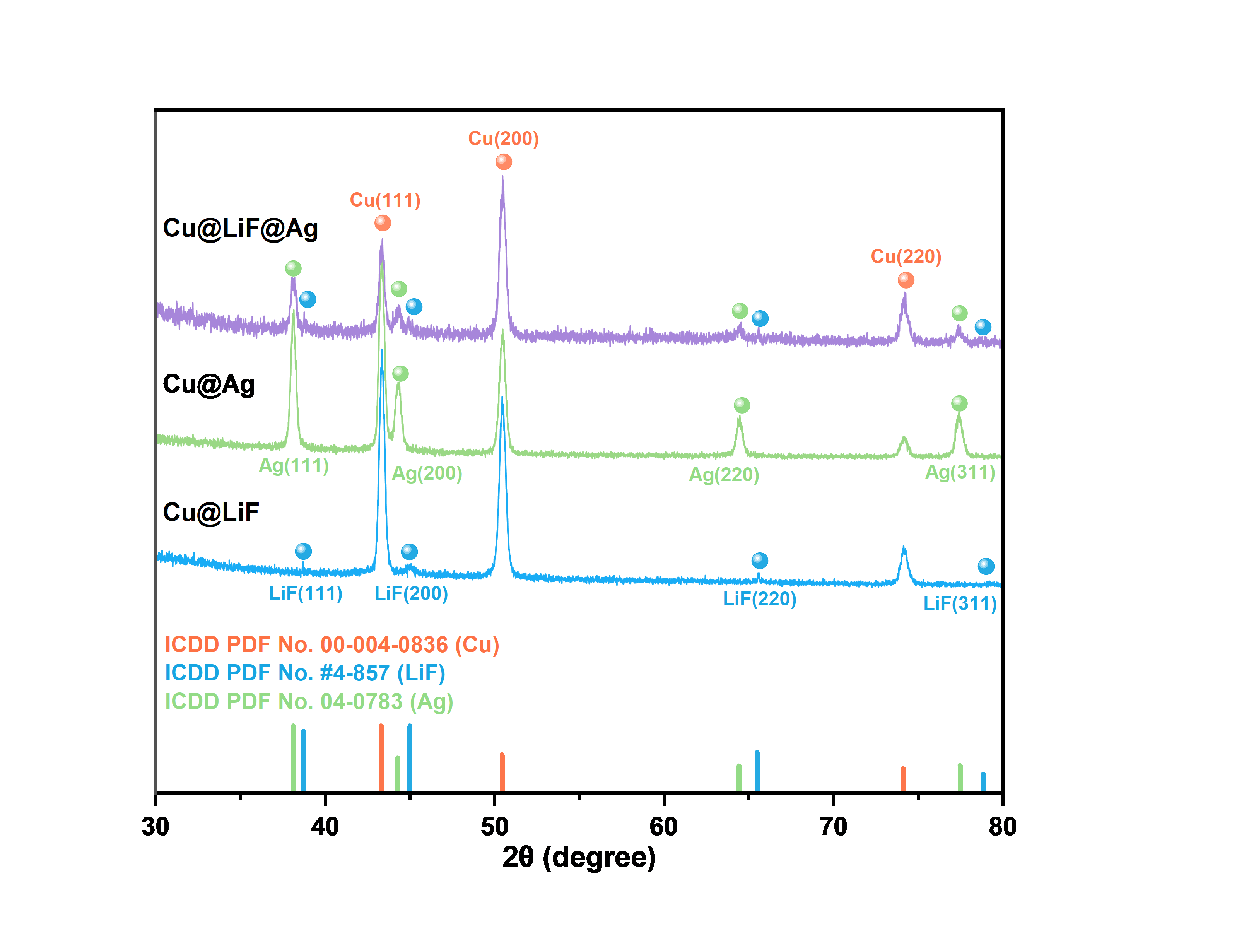


**Figure S2.** GIXRD patterns of the Cu@LiF, Cu@Ag, and Cu@LiF@Ag substrates.
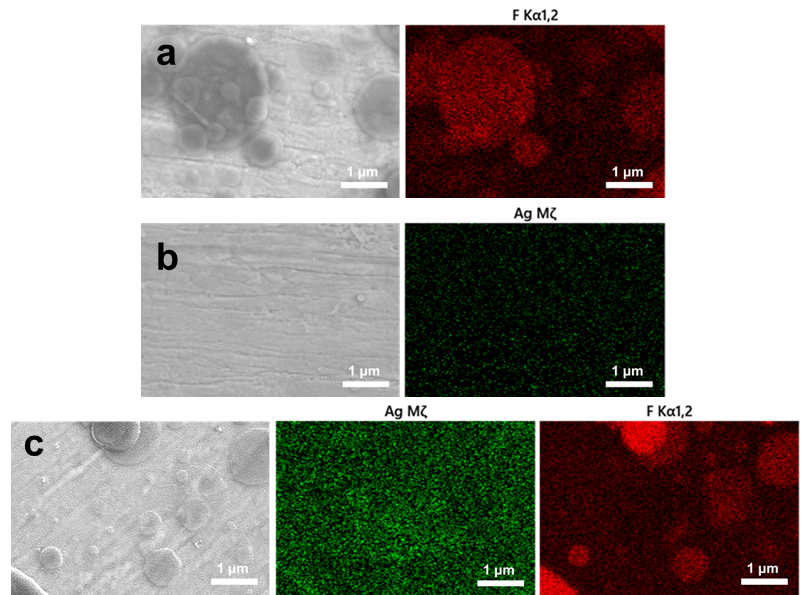


**Figure S3.** SEM images and EDS mapping of the (a) Cu@LiF, (b) Cu@Ag and (c) Cu@LiF@Ag substrates.


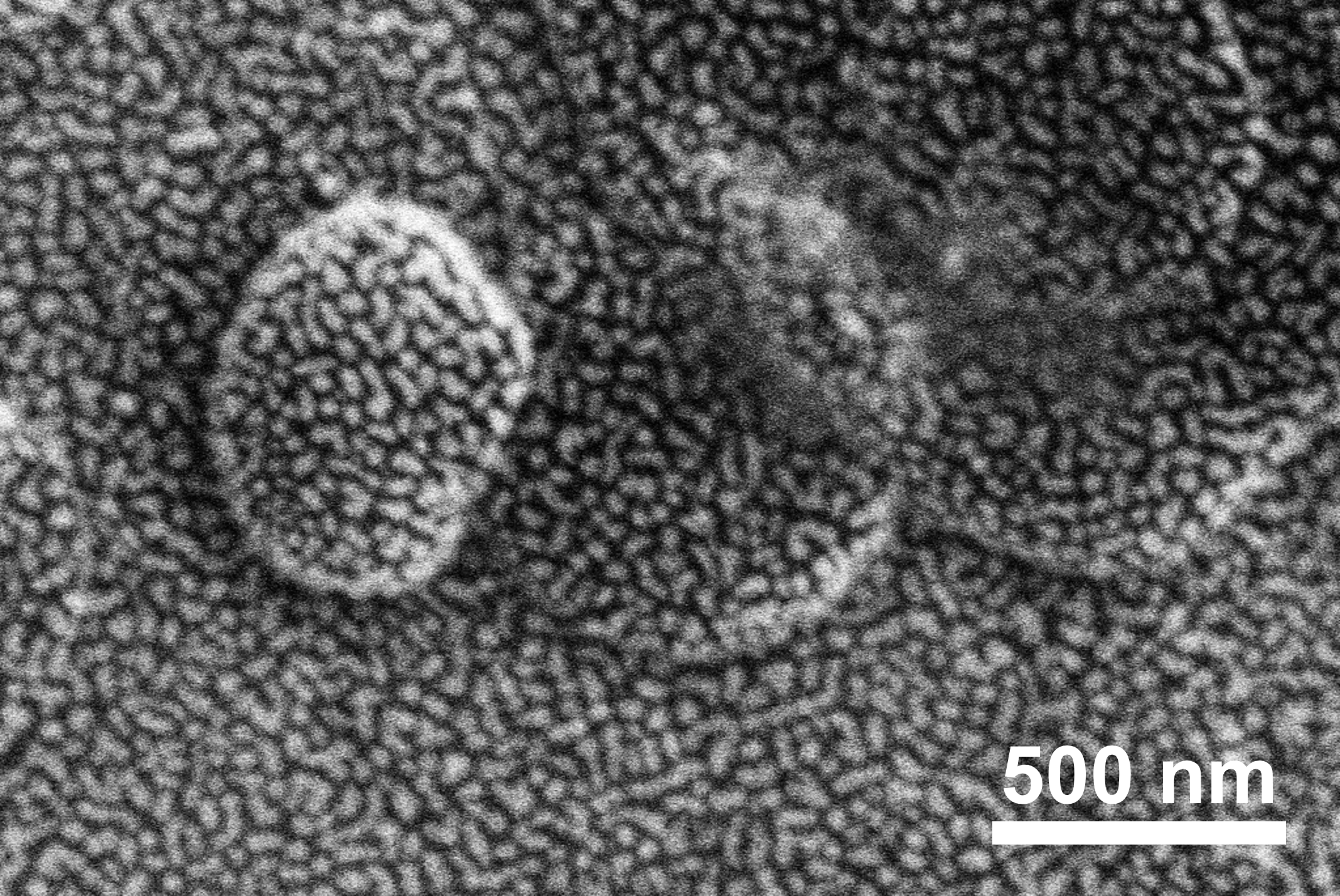


**Figure S4.** Morphology of a Cu@LiF@Ag modified substrate obtained using a longer deposition time of Ag (15 min), showing elongated Ag NPs in contrast to Figure 1e.


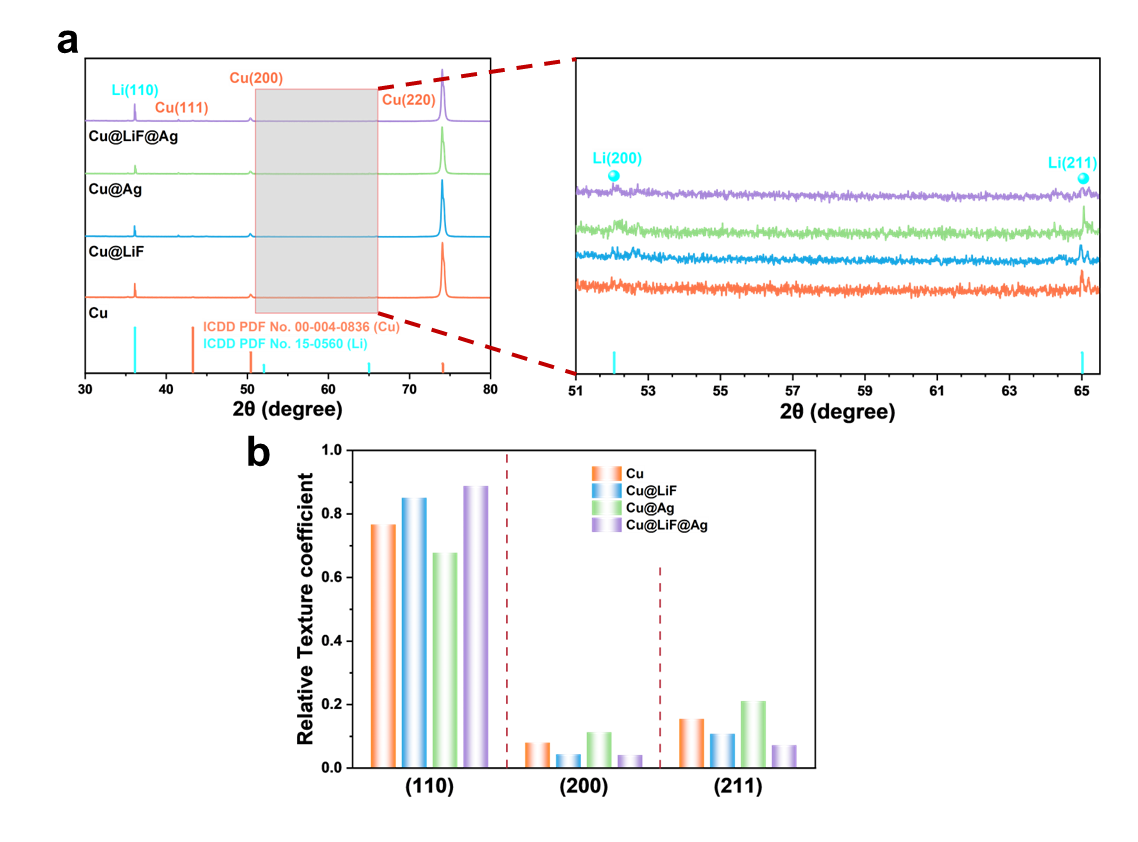


**Figure S5.** (a) XRD patterns of Li deposits on the pristine Cu, Cu@LiF, Cu@Ag and Cu@LiF@Ag substrates. (b) A comparison of the RTC calculated from the XRD patterns.


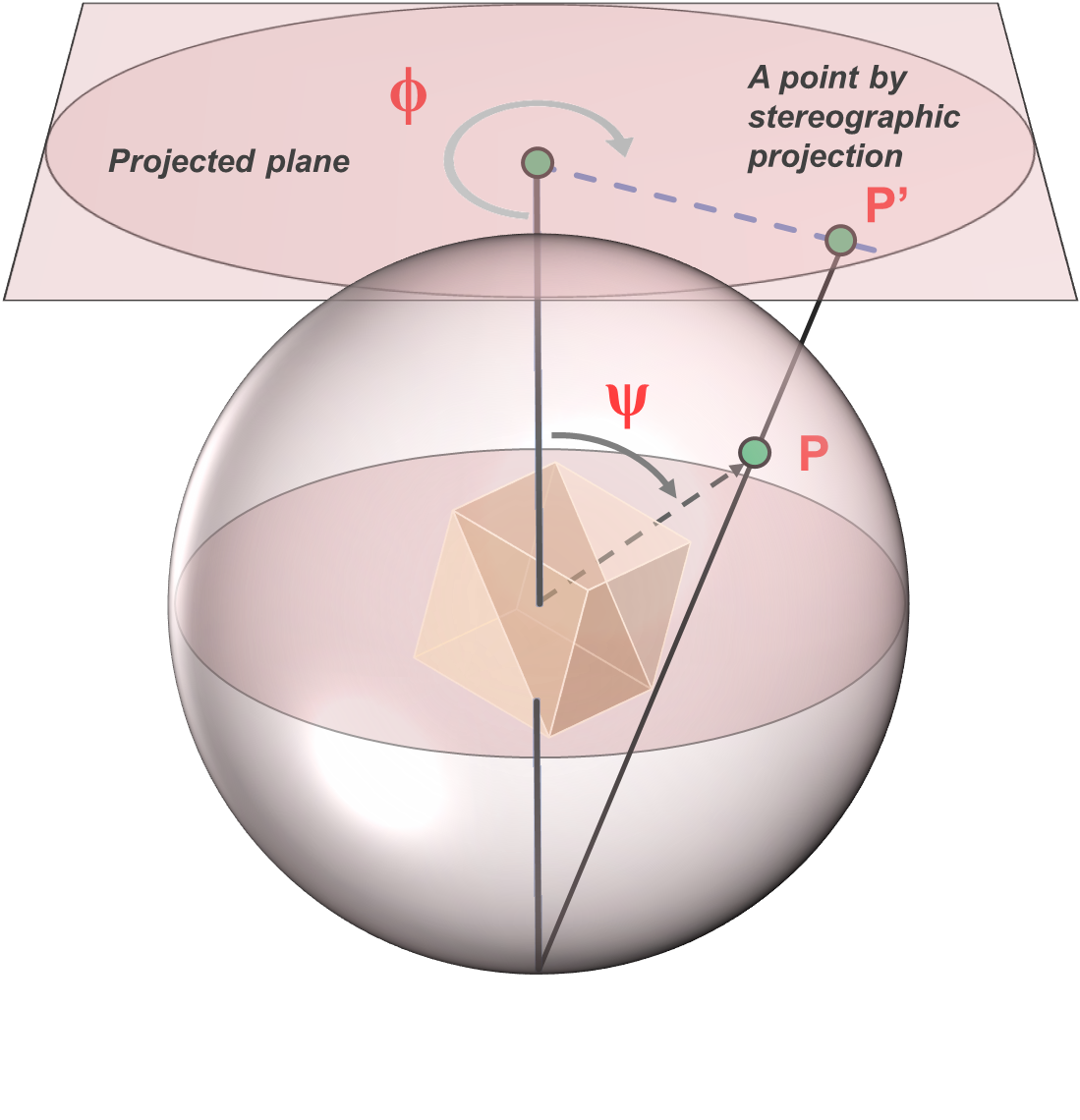


**Figure S6.** A schematic illustration of the pole-figure measurement by stereographic projection at varying rotation angle *ϕ* and tilting angle *ψ*.


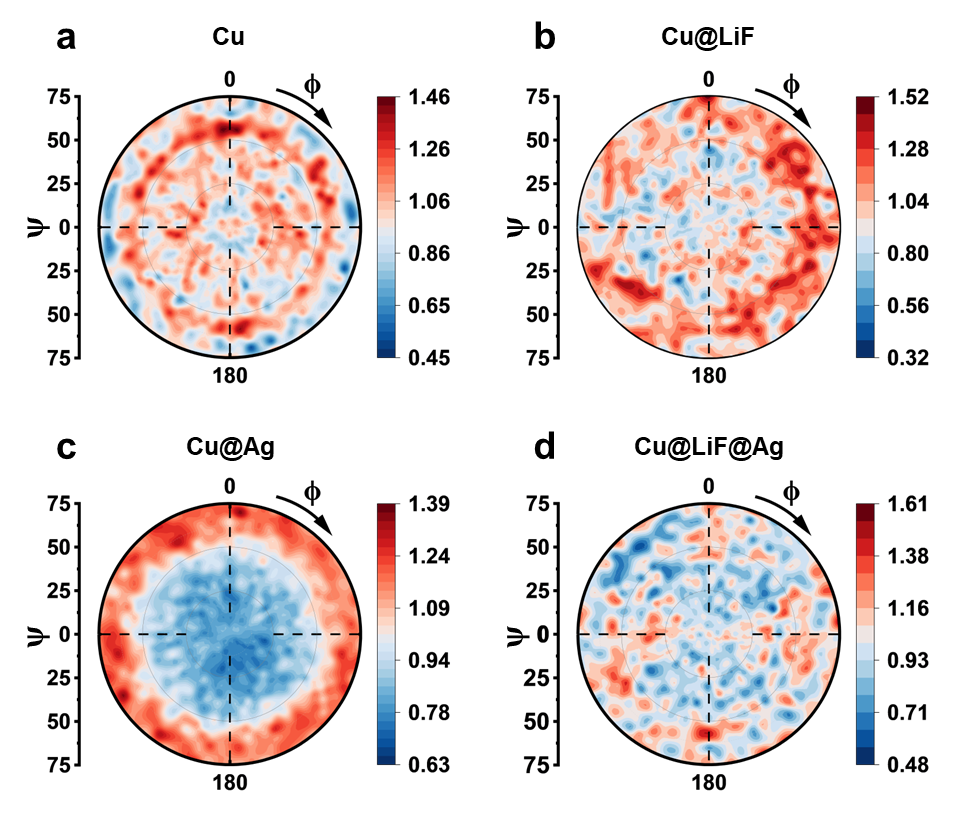


**Figure S7.** The (211) pole figures of 10 mAh cm^−2^ Li deposition on (a) pristine Cu, (b) Cu@LiF, (c) Cu@Ag, and (d) Cu@LiF@Ag.
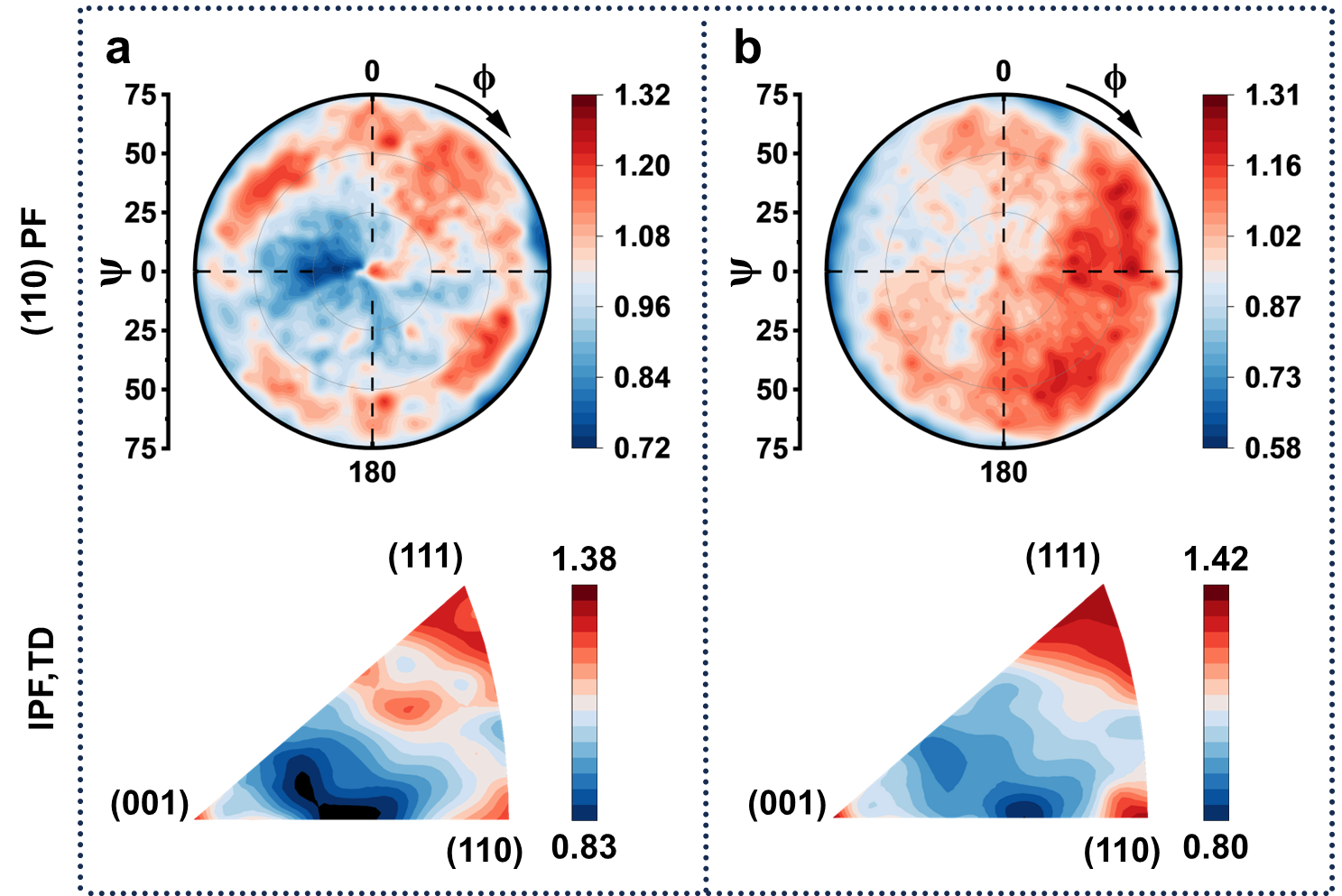


**Figure S8.** The (110) Pole figures (PF) and inverse pole figures (IPF) obtained from XRD for 10 mAh cm^−2^ Li deposition on (a) E-Cu and (b) E-Cu@LiF@Ag.


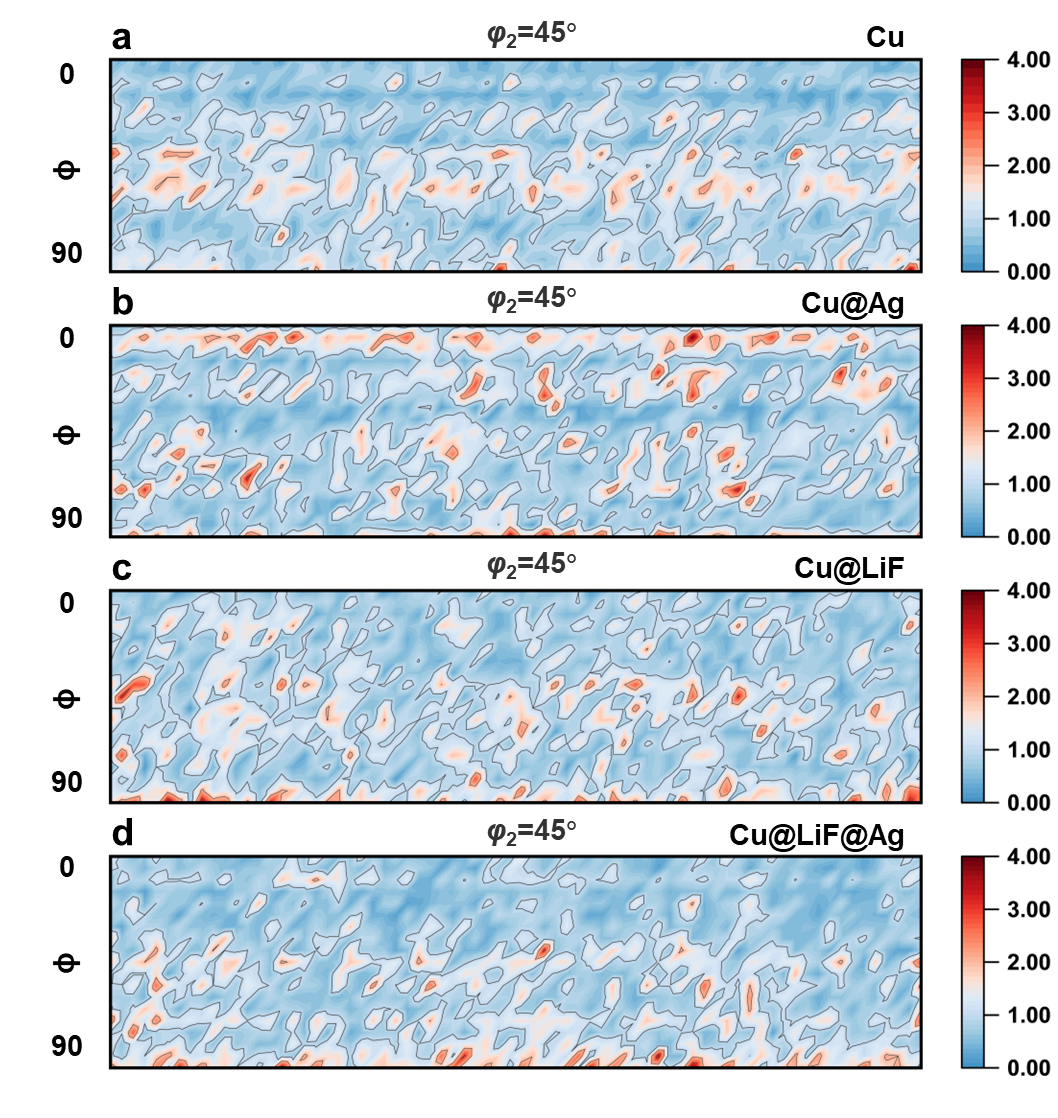


**Figure S9.** The *φ*_2_=45° sections of ODFs of 10 mAh cm^−2^ Li deposition on (a) pristine Cu, (b) Cu@LiF, (c) Cu@Ag, and (d) Cu@LiF@Ag.


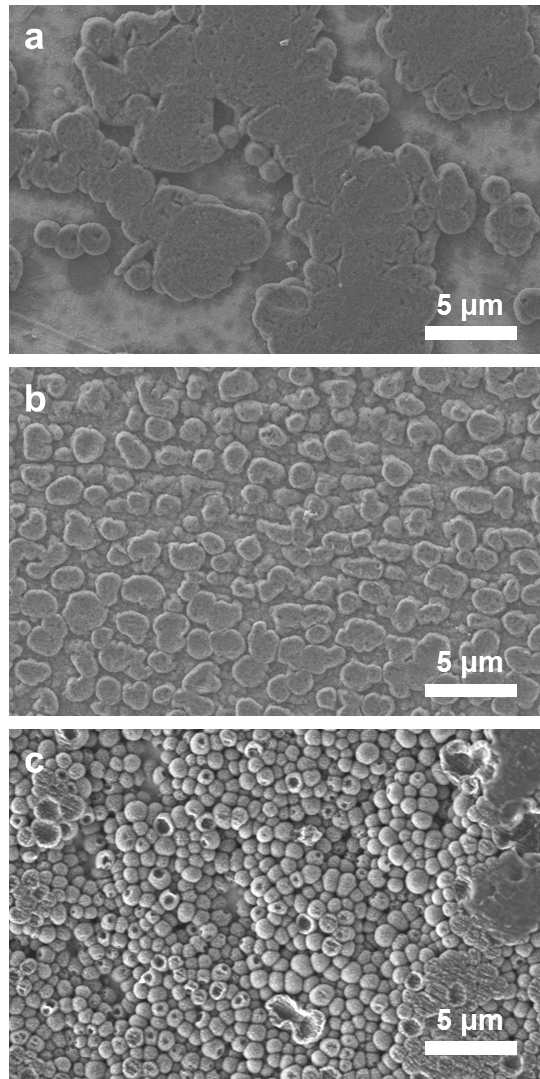


**Figure S10.** SEM morphologies of Li deposited on (a) Cu@LiF, (b) Cu@Ag, and (c) Cu@LiF@Ag at the low capacity of 0.2 mAh cm^-2^, which corresponds to nucleation and early growth.


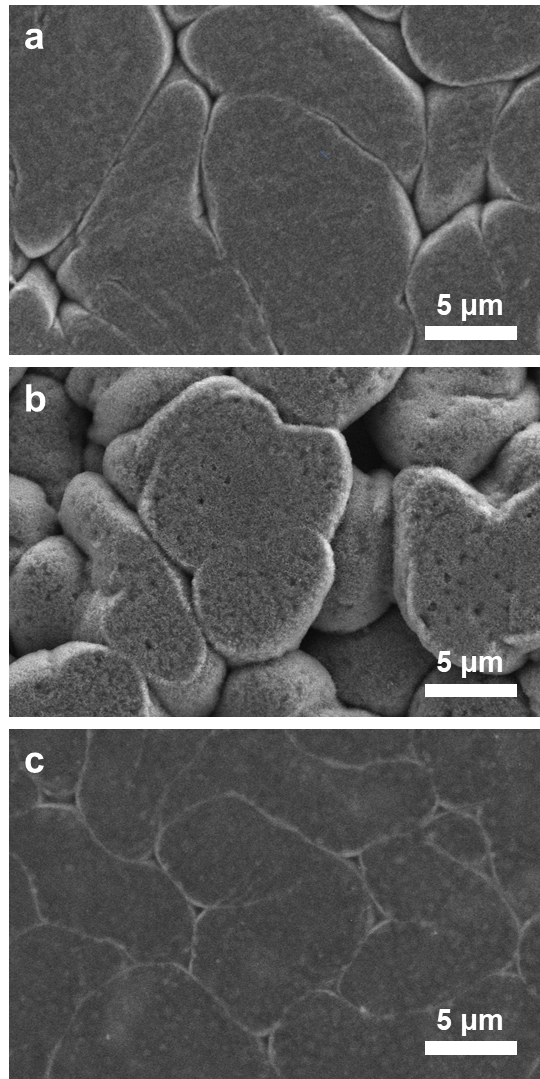


**Figure S11.** SEM morphologies of Li deposited on (a) Cu@LiF, (b) Cu@Ag, and (c) Cu@LiF@Ag at the intermediate capacity of 5 mAh cm^-2^.

**
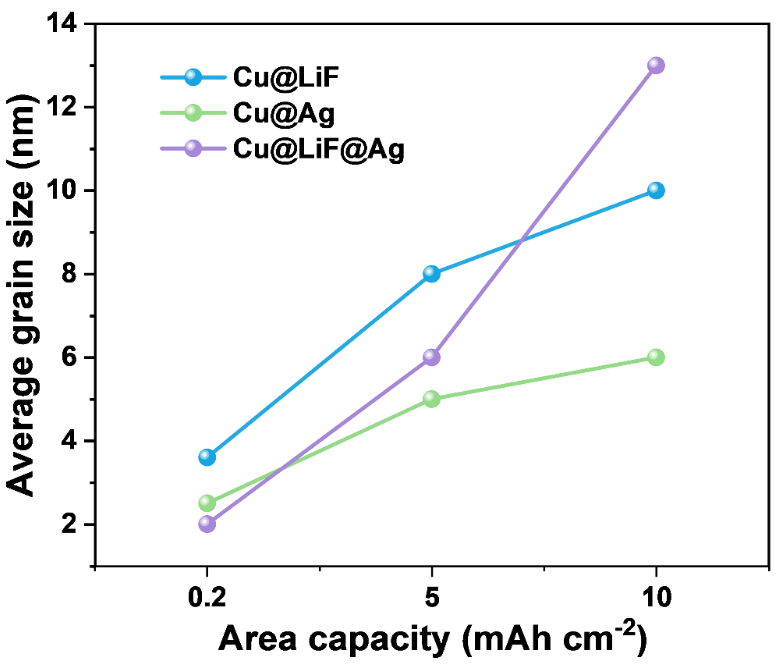
**

**Figure S12.** The evolution of average grain size as a function of deposition capacity on different modified substrates.

**
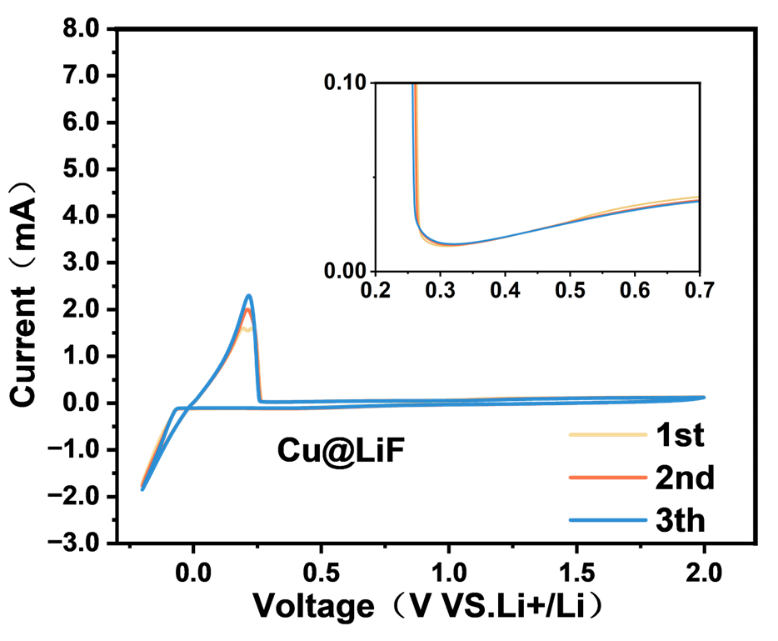
**

**Figure S13.** CV profiles of the Cu@LiF substrate recorded with a scan rate of 1 mV s^−1^. The inset shows a close-up of the anodic scan, where a distinct oxidation peak due to Li dealloying is seen for the Cu@Ag substrate, while being absent for Cu@LiF and Cu@LiF@Ag substrates.

**
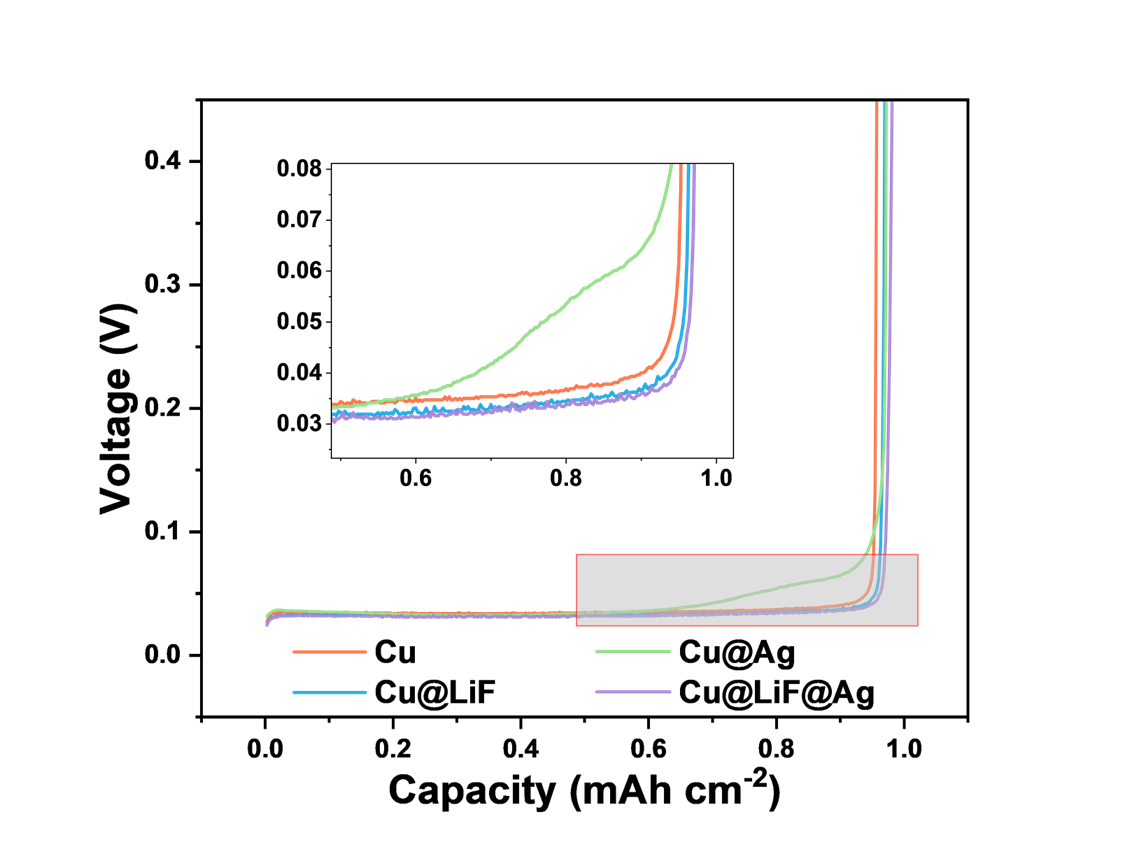
**

**Figure S14.** Voltage profiles of galvanostatic Li stripping at 1 mA cm^−2^.


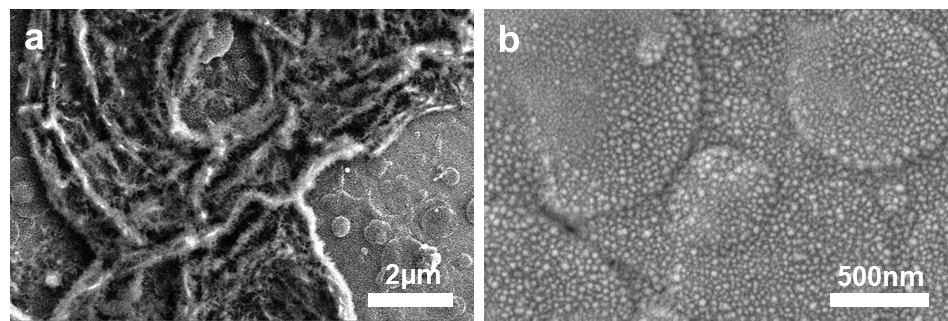


**Figure S15.** SEM images of the Cu@LiF@Ag substrate after 40 cycles of Li plating/stripping in the Li||Cu cell: (a) formation of the SEI layer seen under a low magnification, (b) integrity of the LiF@Ag heterostructure seen under a high magnification.


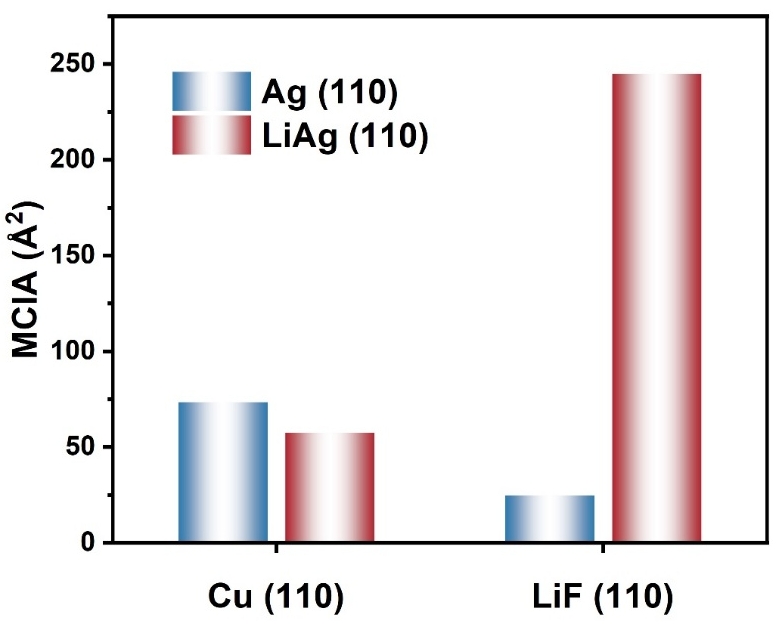


**Figure S16.** Lattice mismatch of Cu(110) and LiF(110) with Ag(110) and LiAg(110). The transformation from a Cu(110)/Ag(110) interface to a Cu(110)/LiAg(110) interface leads to a decrease in the MCIA value from 73.44 to 57.64. In contrast, the transformation of a LiF(110)/Ag(110) interface to a LiF(110)/LiAg(110) interface is hindered by a sharp increase in the MCIA value from 24.48 to 244.96.


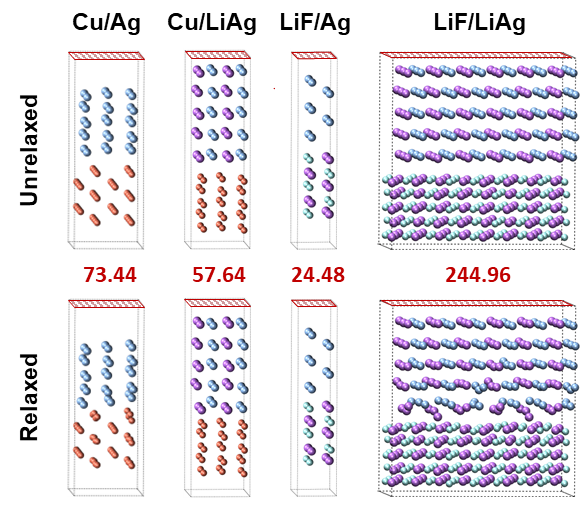


**Figure S17.** Interface models of Cu(110) and LiF(110) with Ag(110) and the β phase LiAg(110) for the calculation of binding energy, interface energy and strain energy. The red dashed lines mark the MCIA in each model. The variations in MCIA and the different bond formation properties lead to different levels of atomic rearrangement at the interface. After relaxation, severe lattice distortions occurred at the Cu/Ag and LiF/LiAg interfaces, consistent with their higher strain energies in Table 1 of the main text.


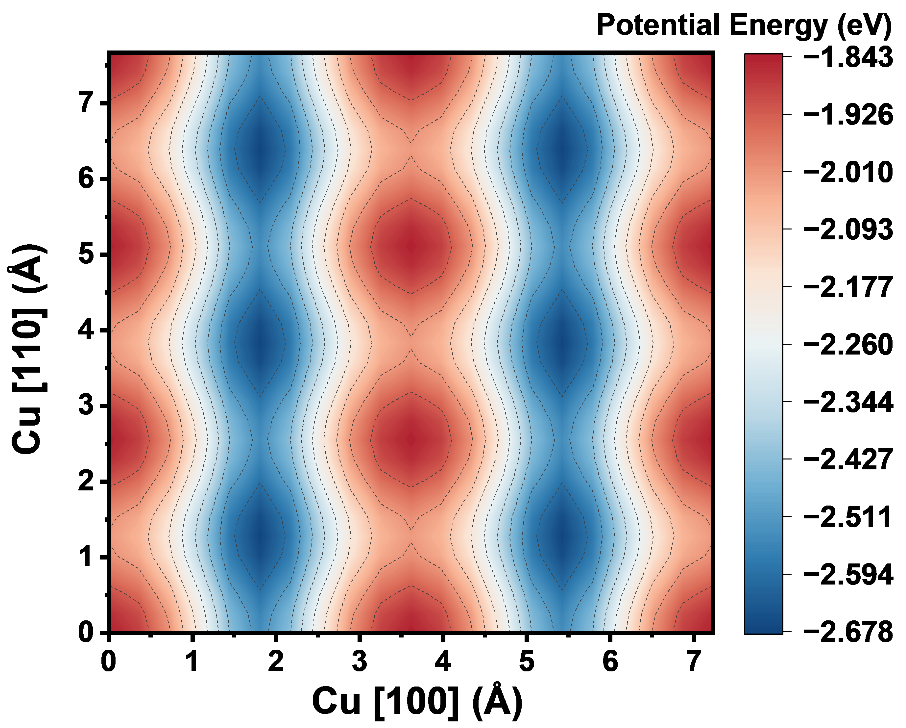


**Figure S18.** The potential energy surface of Li adatoms on Cu(110).


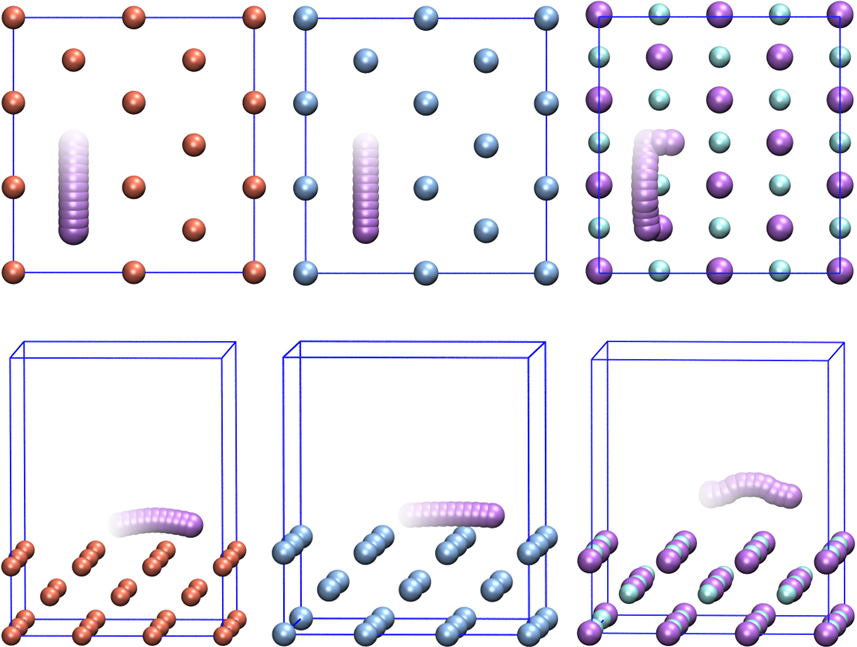


**Figure S19.** Li adatom diffusion paths on (a) Cu(110), (b) Ag(110), and (c) LiF(110) from one stable adsorption site to another. The significantly higher diffusion barrier on LiF(110) is reflected by the curvature of the diffusion path and the relative height of the Li adatom to the slab in the transition state.


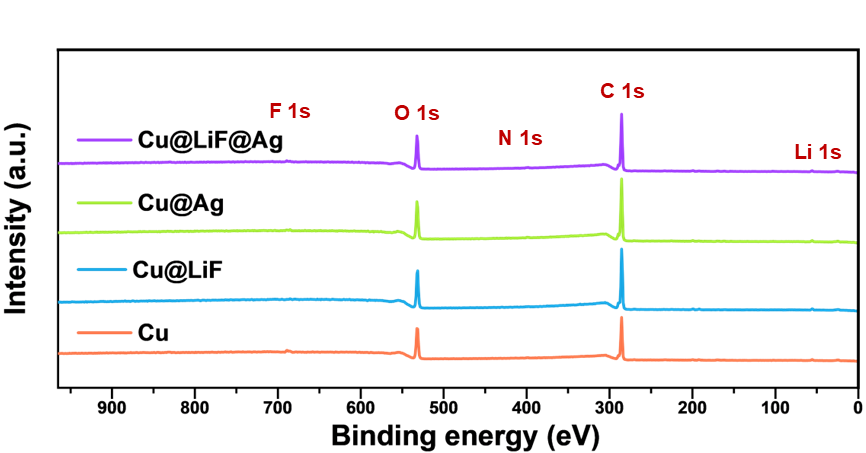


**Figure S20.** XPS survey spectra of the SEI formed on the four different substrates after 15 cycles of Li plating/stripping at 1 mA cm^−2^ and 1 mAh cm^−2^.


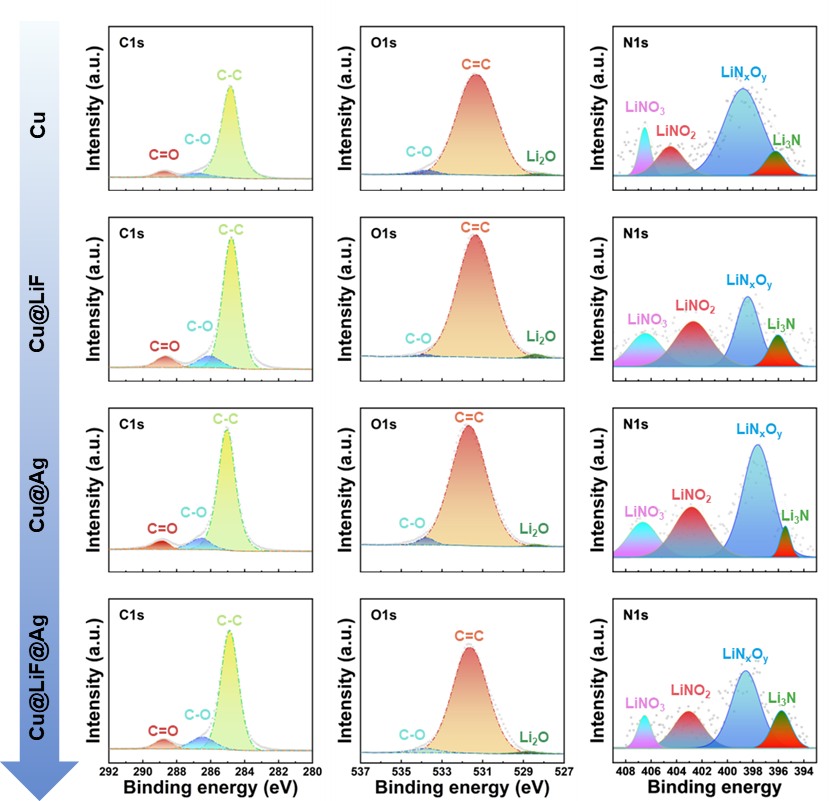


**Figure S21.** XPS C1s, O1s and N1s spectra of the SEI formed on the four different substrates after 15 Li plating/stripping cycles at 1 mA cm^−2^ and 1 mAh cm^−2^.


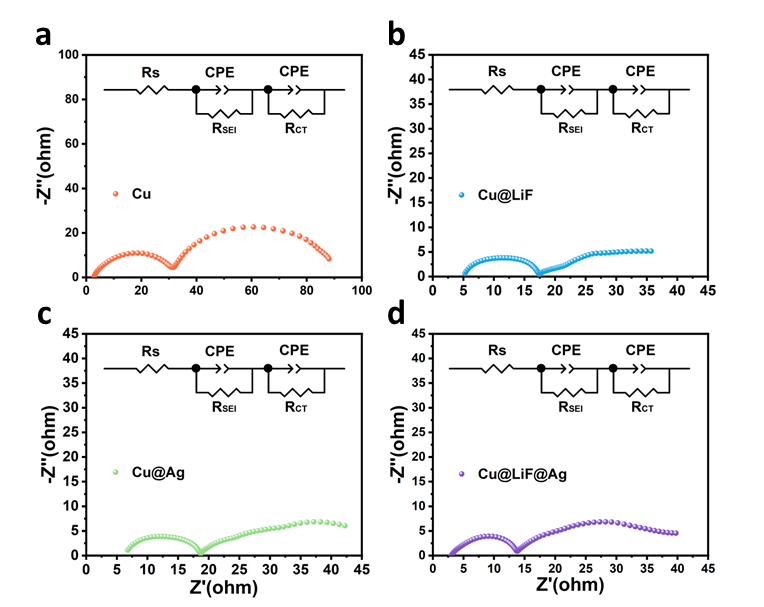


**Figure S22.** Electrochemical impedance spectroscopy (EIS) of the (a) Cu, (b) Cu@LiF, (c) Cu@Ag and (d) Cu@LiF@Ag substrates after 15 cycles of Li plating/stripping.


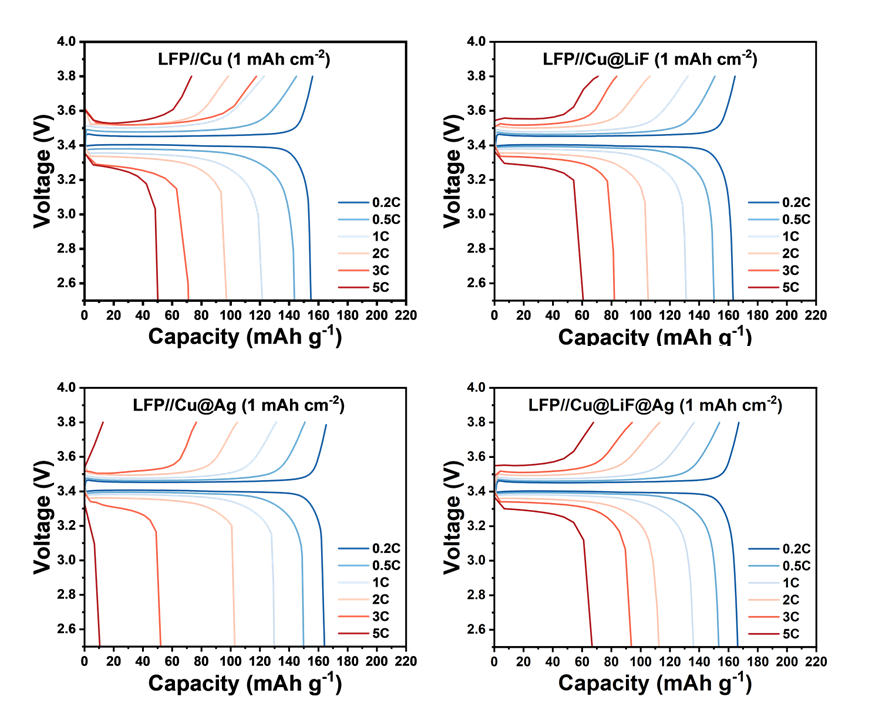


**Figure S23.** Charge and discharge profiles of the LiFePO_4_ full cells containing the four substrates at different C rates.


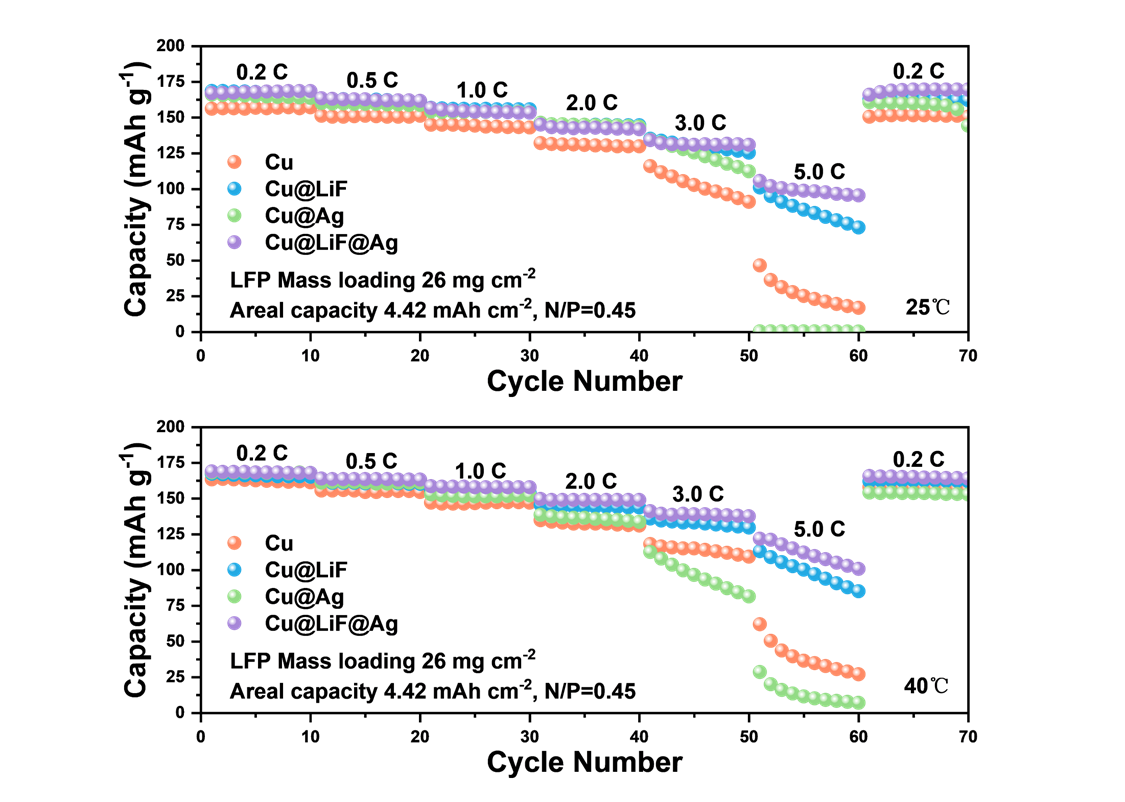


**Figure S24.** High-rate performance of LiFePO_4_ full cells with higher positive capacity at 25 ℃ and 40 ℃.

**Supplementary Tables**

**Table S1.** Comparison of the lattice mismatch for potential materials used as the buffer layer for Li epitaxy on the Cu substrate.

| **Material^*^** | **Li (110) epitaxial layer^**^**  (Å^2^) | **Cu(110) substrate^**^**  (Å^2^) |
| --- | --- | --- |
| Ni (110)  InP (110)  Au (110)  **Ag (110)**  MgO (110)  **LiF (110)** | 16.61  20.34  49.82  **49.82**  49.82  **49.82** | 156.63  147.97  73.82  **73.44**  76.87  **70.74** |

^*^A suitable buffer layer material should exhibit low lattice mismatch with both the Cu substrate and the Li epitaxial layer.

^**^Calculated using the minimal coincident interface area (MCIA), a smaller value means a lower lattice mismatch.

**Table S2.** A comparison of performance between this study and those reported by recent works on modified Cu substrates for Li metal anodes.

| Material | Li-Cu Half cell | | Full cell | | | Ref.^*^ |
| --- | --- | --- | --- | --- | --- | --- |
|  | ACE [%] | CE [%]  (Cycle life and parameter) | Cathode capacity  [mAh cm^-2^] | Rate performance  [mAh g^-1^] (C rate) | Cycle life  (N/P ratio; C rate) |  |
| PVDF | —— | 98.4  (100; 1.0 mA cm^-2^, 1.0 mAh cm^-2^) | 1.75 | 148.2 (0.5)  133.6 (1.0) | 250 (11.4; 1.0) | Ref. [4] |
| Ni-Mg | —— | 98.4  (160; 1.0 mA cm^-2^, 1.0 mAh cm^-2^) | 1.25 | —— | 400 (7.0; 2.0) | Ref. [5] |
| In_3_Li_13_ | —— | 95.0  (100; 0.5 mA cm^-2^, 0.5 mAh cm^-2^) | 0.54 | —— | 350 (7.0; 1.0) | Ref. [6] |
| Sulfurized  CuNW | —— | 99.0  (400; 0.5 mA cm^-2^, 0.5 mAh cm^-2^) | 0.71 | —— | 200 (7.0; 1.0) | Ref. [7] |
| Ag@Zr-DMBD | 99.3 | 99.0  (400; 2.0 mA cm^-2^, 2.0 mAh cm^-2^) | 0.71 | 159.8 (1.0) | 1000 (6.8; 1.0) | Ref. [8] |
| Au-LiF | 99.2 | 98.8  (160; 0.5 mA cm^-2^, 1.0 mAh cm^-2^) | 0.34 | 152 (0.5) | 130 (22.7; 0.5) | Ref. [9] |
|  |  |  |  | 145 (1.0) |  |  |
|  |  |  |  | 117 (5.0) |  |  |
| GaInSn  liquid metal | 99.4 | 99.2  (30; 0.5 mA cm^-2^, 5.0 mAh cm^-2^) | 5.00 | —— | 50 (2.0; 0.2) | Ref. [10] |
| This work | 99.5 | 99.1  (180; 1.0 mA cm^-2^, 1.0 mAh cm^-2^) | 4.42 | 153.4 (0.5) | 220 (0.49; 0.5) |  |
|  |  |  |  | 136.1 (1.0) |  |  |
|  |  |  |  | 70.02 (5.0) |  |  |

^*^References of the Supporting Information.

**References**

[1] a) J. P. Perdew, K. Burke, M. Ernzerhof, *Phys. Rev. Lett.* **1997**, 78, 1396; b) J. VandeVondele, J. Hutter, *J. Chem. Phys.* **2007**, 127, 114105, 114105; c) T. D. Kühne, M. Iannuzzi, M. Del Ben, V. V. Rybkin, P. Seewald, F. Stein, T. Laino, R. Z. Khaliullin, O. Schütt, F. Schiffmann, D. Golze, J. Wilhelm, S. Chulkov, M. H. Bani-Hashemian, V. Weber, U. Borstnik, M. Taillefumier, A. S. Jakobovits, A. Lazzaro, H. Pabst, T. Müller, R. Schade, M. Guidon, S. Andermatt, N. Holmberg, G. K. Schenter, A. Hehn, A. Bussy, F. Belleflamme, G. Tabacchi, A. Glöss, M. Lass, I. Bethune, C. J. Mundy, C. Plessl, M. Watkins, J. VandeVondele, M. Krack, J. Hutter, *J. Chem. Phys.* **2020**, 152, 194103, 194103.

[2] S. Grimme, S. Ehrlich, L. Goerigk, *J. Comput. Chem.* **2011**, 32, 1456.

[3] G. J. Martyna, M. E. Tuckerman, *J. Chem. Phys.* **1999**, 110, 2810.

[4] Y. Wang, F. Xu, E. H. Ang, L. Yang, T. Cui, H. Sun, M. Zhang, T. Yang, H. Zhang, J. Zhu, Y. Hu, *Angew. Chem. Int. Ed****.*** **2025**, 64, e202500323.

[5] Q. Yin, Q. Liu, Y. Liu, Z. Qu, F. Sun, C. Wang, X. Yuan, Y. Li, L. Shen, C. Zhang, Y. Lu, *Adv. Mater.* **2024**, 36, 2404689.

[6] S. Liu, Y. Ma, Z. Zhou, S. Lou, H. Huo, P. Zuo, J. Wang, C. Du, G. Yin, Y. Gao, *Energy Storage Mater.* **2020**, 33, 423.

[7] P. Zou, C. Wang, J. Qin, R. Zhang, H. L. Xin, *Energy Storage Mater.* 2023, 58, 176.

[8] X. Li, Y. Su, Y. Qin, F. Huang, S. Mei, Y. He, C. Peng, L. Ding, Y. Zhang, Y. Peng, Z. Deng, *Adv. Mater.* **2023**, 35, 2303489.

[9] C. Li, Y. Li, Y. Yu, C. Shen, C. Zhou, C. Dong, T. Zhao, X. Xu, *ACS Appl. Mater. Interfaces* **2022**, 14, 19437.

[10] L. Lin, L. Suo, Y.-s. Hu, H. Li, X. Huang, L. Chen, *Adv. Energy Mater.* **2021**, 11, 2003709, 2413420.
